# Advances in the Development of Gradient Scaffolds Made of Nano-Micromaterials for Musculoskeletal Tissue Regeneration

**DOI:** 10.1007/s40820-024-01581-4

**Published:** 2024-11-27

**Authors:** Lei Fang, Xiaoqi Lin, Ruian Xu, Lu Liu, Yu Zhang, Feng Tian, Jiao Jiao Li, Jiajia Xue

**Affiliations:** 1 Beijing Laboratory of Biomedical Materials, Beijing University of Chemical Technology, Beijing, 100029 People’s Republic of China; 2 State Key Laboratory of Organic-Inorganic Composites, Beijing University of Chemical Technology, Beijing, 100029 People’s Republic of China; 3 School of Biomedical Engineering, Faculty of Engineering and IT, University of Technology Sydney, Sydney, NSW 2007 Australia

**Keywords:** Gradient scaffolds, Musculoskeletal tissues, Advanced manufacturing, Biomaterials, Tissue regeneration

## Abstract

The intricate hierarchical structure of musculoskeletal tissues, including bone and interface tissues, necessitates the use of complex scaffold designs and material structures to serve as tissue-engineered substitutes. This has led to growing interest in the development of gradient bone scaffolds with hierarchical structures mimicking the extracellular matrix of native tissues to achieve improved therapeutic outcomes. Building on the anatomical characteristics of bone and interfacial tissues, this review provides a summary of current strategies used to design and fabricate biomimetic gradient scaffolds for repairing musculoskeletal tissues, specifically focusing on methods used to construct compositional and structural gradients within the scaffolds. The latest applications of gradient scaffolds for the regeneration of bone, osteochondral, and tendon-to-bone interfaces are presented. Furthermore, the current progress of testing gradient scaffolds in physiologically relevant animal models of skeletal repair is discussed, as well as the challenges and prospects of moving these scaffolds into clinical application for treating musculoskeletal injuries.

## Highlights

- This review highlights the gradient variations in the structural composition of musculoskeletal tissues and comprehensively examines recent progress in the fabrication and application of biomimetic gradient scaffolds for musculoskeletal repair.
- The challenges and prospects of gradient scaffolds for clinical application are discussed.

## Graphical abstract

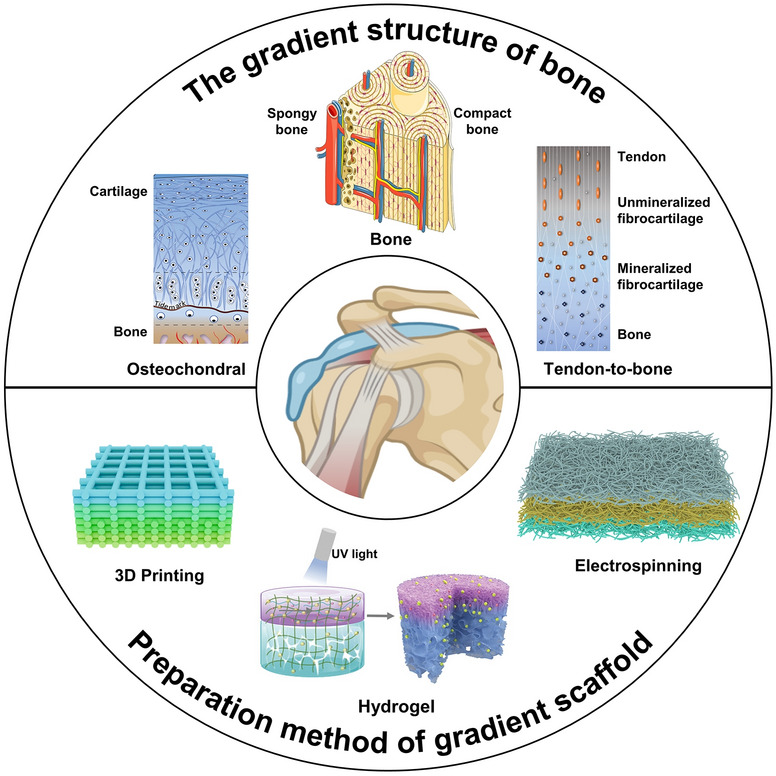

## Introduction

Exacerbated by a globally aging population, the treatment of musculoskeletal conditions arising from trauma and chronic diseases is becoming an increasingly important healthcare concern [1]. Although natural bone tissue can self-repair for injuries with a critical threshold of approximately 2 cm, complete healing is usually only possible for small or confined areas of bone loss. If the defect area is complex or exceeds this critical threshold, surgical intervention is necessary to facilitate the healing process. Bone transplantation is the primary surgical method used for treating bone defects [2, 3]. Currently, the categories of clinically used materials for bone repair include autologous, allogeneic, and artificial bone grafts. However, as the clinical gold standard, the use of autologous bone is constrained by supply shortage, donor site injury, and additional complications, while the alternative use of allogeneic bone experiences problems of poor tissue integration and vascularization along with a potential risk of immune rejection or infection. Using tissue engineering strategies, artificial bone scaffolds have recently emerged as an improved approach to bone repair. They offer the advantages of flexible structural design, the capacity for mass production, and the potential to incorporate biologically active factors, drugs, or external stimuli based on individual requirements. While few products have been translated into clinical applications, the advantages of artificial bone scaffolds have made them a mainstream trend in current research into bone repair strategies [4, 5].

Bone is a highly dense and complex calcified tissue composed of organic protein, inorganic minerals, and various cell types [6]. Natural bone tissue and bone-containing interface tissues often exhibit a combination of structural and compositional gradients, with discrete or continuous change in properties depending on the specific tissue/interface region, demonstrating a high level of hierarchical organization. In addition to the difficulties of repairing bone tissue alone, pathologies that occur at the interface between bone and other connective tissues pose significant challenges to successful repair, with chronic impacts on human health and quality of life, such as rotator cuff tears, patellar tendon injuries, and osteochondral defects [7]. Clinically, injuries at the tendon–bone interface are frequently treated with suture anchors and tendon transposition, but these procedures are prone to the postoperative development of scar tissue, which is mechanically inferior to the normal tendon–bone insertion point and may lead to poor recovery or even recurrence. The failure rate of surgical treatment for tendon–bone injuries has been reported to reach 20%–95% [8–10]. For osteochondral injuries, the most commonly used clinical procedures include mosaicplasty [11], subchondral bone drilling [12], and microfracture [13]. However, these methods frequently lead to the formation of fibrocartilaginous or scar tissue, resulting in joint resurfacing by tissues that are mechanically inferior to healthy articular cartilage or do not integrate well with surrounding tissues, thereby predisposing the joint to degenerative conditions such as osteoarthritis [12, 14]. The successful regeneration of musculoskeletal tissue, both in large bone defects and also as an essential component of bone-containing interface tissues, is therefore critical to the long-term healing of musculoskeletal injuries and has prompted increasing research attention in the development of artificial bone scaffolds.

Although a variety of strategies have been reported for constructing bone scaffolds, their effectiveness at inducing satisfactory healing has been suboptimal as these scaffold designs mostly do not match the native gradients seen in the majority of musculoskeletal tissues [15]. Gradients are an inherent feature of biological structures, with crucial functions in tissue physiology and development. With advances in design and manufacturing technologies, the biological gradients found in different types of musculoskeletal tissues have been increasingly used as biomimetic inspirations for constructing artificial bone scaffolds [14]. Arising from different fabrication techniques, gradient bone scaffolds are generally categorized into layered gradient and continuous gradient. The former scaffold design is usually divided into discrete layers, each with a different structure and active ingredient. In the latter, the change in structure and active ingredients of the scaffold form a continuous transition, more closely replicating native tissue structures. Either way, gradient bone scaffolds matching the compositional and/or structural gradients seen in native musculoskeletal tissues can lead to improved material–tissue integration and regeneration of anatomically and physiologically similar bone and bone-containing interface tissues [16]. They may also enable better reconstruction of the intricate mechanical environments that form a critical part of musculoskeletal tissue function. Additionally, biochemical concentration gradients may be incorporated into scaffolds, such as a growth factor gradient to enable the recruitment of endogenous stem cells to the defect site to aid tissue regeneration [17]. The development of gradient scaffolds for bone and interfacial tissue regeneration has been partially captured in a few recent reviews, some of which have discussed specific fabrication techniques such as hydrogels [18] and additive manufacturing (biofabrication) [19], while others have focused on specific tissue types such as osteochondral tissue [12] and anterior cruciate ligament [20]. In this review, we comprehensively summarize and critically analyze the latest research advances on gradient artificial bone scaffolds designed for the regeneration of different types of musculoskeletal tissues, including bone, osteochondral tissue, and the tendon–bone interface. We also present the current evidence on different fabrication strategies used to realize these gradient scaffold structures, including electrospinning, additive manufacturing (biofabrication), and hydrogel fabrication methods. The preclinical effectiveness of gradient bone scaffolds applied in animal models of musculoskeletal injuries is discussed, giving insights into their potential for future clinical application. Our review provides an up-to-date summary of the most impactful developments in this exciting area of research and offers perspectives on the status and prospects of translating gradient bone scaffolds from a laboratory setting into clinical practice.

## Bone Tissue Engineering and Scaffold-Based Strategies

Bone tissue engineering is a cutting-edge frontier in biomedical research and regenerative medicine. Harnessing the power of advanced biomaterials, stem cells, and innovative engineering approaches, the field of bone tissue engineering aims to revolutionize the treatment of bone and musculoskeletal injuries, including those involving osteochondral tissue and the tendon-to-bone interface. The design and fabrication of gradient bone scaffolds provide a biomimetic approach to regeneration, with a number of recent studies highlighted in Table 1, which have been surveyed from the literature in the last 5 years. Studies reporting new designs of gradient scaffolds have been categorized by application into cortical and cancellous bone, osteochondral tissue, and tendon-to-bone interface, along with a summary of the corresponding method of fabrication, design of scaffold gradient architecture, selection of biomaterials, and outcomes of biological evaluation in vivo findings. The structural and compositional features of native tissues and their extracellular matrix (ECM) replicated by gradient scaffold designs are explained in the below sections.

**Table 1:** Scaffold design and type of gradient

Name of scaffold	Fabrication method	Cells	Materials used and biomimetic scaffold architecture	Characterizations—morphology and mechanical properties	In vitro testing (cell type)	In vivo testing	Ref
*Bone*							
Layered gradient—drug-loaded 3D bone scaffolds with radial gradient porosity	FDM	N/A	Material used: PLA loaded with ibuprofen Radial gradient porosity: Three infill zones designed with gyroid infill with varying infill densities: Outer zone: 100% infill, mimicking cortical bone to provide mechanical strength Transition zone: 40% infill, imitating the porous structure of trabecular bone Core zone: 20% infill, facilitating cell and nutrient flow, resembling the inner bone structure	Pore size: The radial gradient scaffolds (G100-40–20) were designed with a gyroid infill pattern: Outer zone (100% infill): smaller pores Transition zone (40% infill): medium pore size Core zone (20% infill): larger pores (No numerical value for pore size provided) Mechanical properties: G100 (outer zone, smallest pores) provided the highest mechanical strength due to its lower porosity G100-40–20 scaffold (gradient) had compressive strength comparable to cancellous bone (5–10 MPa), balancing both mechanical support and biological function	Cell line: L929 fibroblast cells (seeding density: 10^5^ cells/mL) In vitro results: Scaffolds with radial gradient porosity exhibited increased cell proliferation and drug release but lower mechanical strength due to higher porosity The radial gradient scaffolds (G100-40–20) had better cell proliferation due to the highly porous center, which facilitated cell migration and nutrient diffusion The cumulative drug release from the radial gradient scaffolds was around 50% after 120 h	N/A	[30]
Continuous gradient—radial gradient long bone scaffold (BS-G)	3D continuous extrusion printing	N/A	Materials used: sodium alginate, gelatin, and nano-hydroxyapatite (nHAp) Radial gradient structure: pore size gradually increases from the outer dense structure (mimicking cortical bone) to the inner porous structure (mimicking cancellous bone) nHAp concentration decreases from the outermost layer (3%) to the innermost layer (0%)	Pore size: Gradient in pore size, with smaller pores on the outer layer (mimicking cortical bone) and larger pores on the inner layer (mimicking cancellous bone). The pore size gradually increases from 0.7 mm at the outer layer to 1.5 mm at the innermost layer Mechanical properties: The scaffold demonstrated a compressive strength of 1.00 ± 0.19 MPa, which is sufficient for bone defect repair, as it falls within the range needed to support natural bone healing	Cell line: human umbilical vein endothelial cells (HUVECs) and C57BL/6 mouse BMSCs In vitro results: The scaffold BS-G showed no cytotoxic effects, indicating that it is biocompatible and suitable for cell culture No cytotoxicity observed Supported cell proliferation	Animal model: Sprague–Dawley (SD) rats (male, 6 weeks old) Time length: two-week implantation period Conclusion of In Vivo Results: The histological analysis revealed no significant differences in tissue staining between the experimental group and the control group, further confirming the good biocompatibility of the BS-G scaffold The scaffold was well tolerated in the rat model, with no signs of inflammation or toxicity, indicating its potential for use in bone defect repair applications	[31]
Continuous gradient -Haversian system-like gradient porous scaffold	SLM	N/A	Material used: Ti_6_Al_4_V alloy (titanium alloy) Gradient porous structure: Gradient structure with pore size decreasing from the ends (650 µm) to the center (450 µm) Biomimetic design mimicking the Haversian canal, Volkmann canal, and cancellous bone The peripheral larger pores form Haversian canal-like tunnels, smaller central pores resemble cancellous bone	Pore sizes: Outer region pore size 650 µm, center region pore size: 450 µm, with porosity of approximately 50.77% Mechanical properties (gradient scaffold): Young’s modulus: 10.62 ± 0.45 GPa Compression stress limit: 509.01 ± 4.29 MPa	Cell line: mouse pre-osteoblast cell line MC3T3-E1 In vitro results: The gradient scaffold promoted higher cell adhesion, proliferation, and osteogenic differentiation compared to the uniform scaffold Osteogenesis-related gene expression (ALP, COL1, OCN, RUNX2) and protein expression (OCN, COL1) were significantly higher on the gradient scaffold Increased ALP activity and mineralization Improved cell adhesion, proliferation, and osteogenic activity	Animal model: male New Zealand rabbit (3 kg), bilateral radial bone defects (20 mm in length) Time length: 12 weeks Conclusion of in vivo results: The gradient scaffold showed greater bone ingrowth, improved bone defect bridging, and better osseointegration compared to the uniform scaffold Push-out force (bone scaffold integration strength) was higher in the gradient scaffold (302 N) than in the uniform scaffold (170 N) Histological analysis revealed more bone formation inside the pores of the gradient scaffold, with bone extending deeper into the scaffold The gradient scaffold exhibited enhanced vascularization and bone formation, both in peripheral and central regions of the scaffold	[32]
Continuous gradient—Gradient Voronoi Scaffold	SLM and electron beam melting (EBM)	N/A	Materials used: Ti_6_Al_4_V (Titanium alloy) Radial gradient structure: Irregular porous structure based on Voronoi tessellation with a gradient design, mimicking the trabecular bone structure pore size gradually increases from the outer dense structure (mimicking cortical bone) to the inner porous structure (mimicking cancellous bone) nHAp concentration decreases from the outermost layer (3%) to the innermost layer (0%)	Morphology: irregular polyhedral shapes generated through Voronoi tessellation; gradient density along different axes, porosity of the scaffold designs was around 94.4% Mechanical properties: The scaffold demonstrated improved stability and impact resistance over regular porous structures	N/A	N/A	[29]
*Osteochondral tissue*							
Layered gradient—multilayered silk fibroin composite scaffold	Freeze-drying technique	N/A	Materials: SF Incorporation of HAp in different percentages Three distinct layers: Bone layer: 5 wt% concentrated SF solution and 3% w/v SF-3%HAp nanofibers Intermediate layer: 12 wt% SF solution and 3% w/v SF-1%HAp nanofibers Cartilage layer: 9 wt% SF solution and 3% w/v SF nanofibers	Morphology: Bone layer: 152.06 ± 24.02 μm pore size, 80.38 ± 3.47% porosity Intermediate layer: 76.33 ± 10.22 μm pore size, 68.37 ± 6.47% porosity Cartilage layer: 102.62 ± 12.48 μm pore size, 78.34 ± 3.34% porosity Mechanical properties (compressive modulus): Bone layer: 0.45 ± 0.60 MPa Intermediate layer: 0.28 ± 0.17 MPa Cartilage layer: 0.16 ± 0.34 MPa	Cell Line: Rabbit adipose-derived stem cells (RADSCs) were sourced from 4-month-old New Zealand rabbits In vitro results: Cell viability and proliferation (methylthiazolyldiphenyl-tetrazolium bromide assay, MTT): The MTT assay showed an increase in cell viability and proliferation. The dynamic culture exhibited significantly higher cell proliferation compared to the static culture Gene expression analysis: After 21 days, the expression of osteogenic genes (COL1, OPN, OCN) was significantly upregulated in the bone layer. The expression of chondrogenic genes (COL II; SRY-box transcription factor, SOX9) was significantly higher in the cartilage layer. The expression of hypertrophic gene (COL X) was downregulated in the intermediate layer under dynamic culture Histological analysis: Hematoxylin and eosin (HandE) staining revealed a more homogeneous distribution of RADSCs within the scaffold layers after 7 days of dynamic culture, indicating better cell infiltration compared to static culture. The cell density was higher in all layers	N/A	[46]
Layered gradient—zonal-structured matrices (osteochondral scaffold)	Extrusion-based 3D bioprinting	Cell-laden bioink: The immortalized C28/I2 human chondrocyte cell line	Materials: PLA for the scaffold and alginate hydrogel for cell encapsulation Architecture: The gradient scaffold consists of seven zones with varying porosity and infill density to mimic cartilage and bone	Tri-phasic scaffold: The pore size ranged from larger pores at the top to smaller pores at the bottom, mimicking the transition from cartilage to bone Specific pore sizes were not explicitly detailed for each zone of the gradient scaffold, but the overall pore size range was similar to 109.8 µm to 342.0 µm, showing a gradual transition Mechanical properties: The compressive modulus of the gradient scaffold was the lowest among all configurations: 177.98 MPa ± 44.78 MPa The compressive yield strength was also the lowest: 4.20 MPa ± 1.03 MPa	Cell line: C28/I2 human chondrocyte cell line In vitro results: Cell viability: high cell viability (> 79%) with uniform cell distribution in concurrent bioprinted scaffolds Mechanical properties: biphasic scaffolds showed the highest compressive modulus and yield strength, while gradient scaffolds had the lowest due to the increased number of zones	N/A	[48]
Layered gradient—biomimetic multidirectional scaffolds	Unidirectional freeze casting combined with a lyophilization bonding process	N/A	Materials used: Superficial zone: Col I and HA Osseous zone: Col I and HAp Transition zone: Col I and HA (different ratio from the superficial zone) Architecture: Superficial zone: compressed, highly aligned collagen-HA fibers (~ 125 μm thick) Transition zone: isotropic pore structure mimicking native transition zone architecture (~ 1.4 mm thick) Calcified cartilage zone: A combination of transition zone and osseous zone features (~ 0.6 mm thick) Osseous zone: Vertically aligned sheets of mineralized collagen with a lamellar structure	Pore morphology: Superficial zone: small pores (~ 48 μm) with high anisotropy Transition zone: largest pores (~ 105 μm) with low anisotropy Calcified cartilage zone: Pore size of ~ 88 μm with high anisotropy Osseous zone: pores of ~ 98 μm between lamellae with high anisotropy Mechanical properties: Superficial zone: elastic modulus (E*) of 7.2 kPa Transition zone: E* of 12.7 kPa Calcified cartilage zone: E* of 13.9 kPa Osseous zone: E* of 17.0 kPa	N/A	N/A	[47]
*Tendon–bone interface*							
Continuous gradient—Continuous mineral gradients for tendon-to-bone tissue engineering	A combination of electrospinning and traditional textile manufacturing techniques	N/A	Material used: poly(L-lactide-co-ε-caprolactone) (PLCL), SF, PLA microfiber, and nHA Architecture: The scaffold has a core sheath yarn structure, with a gradient of HAp content (0%, 5%, 10%, and 15%) to simulate the tendon-to-bone interface. The scaffold is anisotropic, with spatial variations in HAp content to mimic the transition from tendon to bone	Morphology: gradient scaffold had a porosity (22 ± 5%) and pore size (122 ± 17 μm) good interconnectivity Mechanical properties: Ultimate tensile strength (UTS) and Young's modulus of weft direction for segments with different HAp contents: UTS ranged from 23.4 ± 2.0 to 34.2 ± 2.4 MPa and Young’s modulus ranged from 193.7 ± 11.9 to 289.3 ± 9.2 MP	Cell line: MC3T3-E1: assess the osteogenic potential of the mineralized segments of the scaffold Rat BMSCs (rBMSCs): used to evaluate the differentiation potential of the scaffold In vitro results: In vitro studies showed that mineralized segments of the scaffold boosted the proliferation of MC3T3-E1 and promoted osteogenic differentiation of rBMSCs The scaffold was able to spatially guide the differentiation of rBMSCs, leading to the formation of neotissue with characteristics similar to the tendon–bone insertion site	N/A	[57]
Continuous gradient -Cocktail-like gradient gelatin/ hyaluronic acid bioimplant (BMSCs + gNC@GH)	Four-layer cocktail-like hydrogel structure by layering different concentrations of NC within the GelMA/HAMA dual-network gel. UV curing for cross-linking the hydrogel layers	BMSCs	Materials used: Gelatin (GelMA) Hyaluronic acid (HAMA) Nanoclay (NC): Laponite, lithium magnesium silicate) BMSCs (for cell encapsulation) Architecture: The scaffold has a four-layer structure with a gradient distribution of NC, designed to mimic the natural tendon–bone interface. Each layer has a different concentration of NC, creating a gradient from low to high NC content	No numerical values were provided for pore morphology and mechanical properties	After 7 days of culture, live-dead staining showed that most BMSCs remained viable, indicating the biocompatibility of the scaffold In vitro results: Cell proliferation: The scaffold supported the proliferation of BMSCs and 3T3-L1 cells Osteogenic differentiation: The gradient distribution of NC in the scaffold promoted a gradient of osteogenic differentiation in BMSCs, as evidenced by increased expression of osteogenic markers (Runx2 and OPN) Gene expression analysis: quantitative real-time PCR (Q-PCR) confirmed the upregulation of osteogenic genes (Runx2 and OPN) and downregulation of adipogenic genes in cells cultured with the scaffold, suggesting its ability to guide cell fate toward osteogenic lineage	Animal model: 8-week-old SD rats weighing 300–400 g, rotator cuff injury In vivo results: Cocktail-like hydrogel was capable of promoting gradual osteogenic differentiation and inhibiting adipogenic differentiation Fibrocartilage regeneration: The scaffold effectively promoted the regeneration of the fibrocartilage layer at the tendon–bone interface, as evidenced by safranin-O and fast green staining Histological analysis: Histopathological staining showed improved fibrocartilage regeneration and reduced fatty infiltration in the BMSCs + gNC@GH group The BMSCs + gNC@GH group exhibited a higher degree of organization of chondrocytes and the presence of mature chondrocytes	[58]
Layered gradient—multilayer nanofiber-reinforced 3D scaffolds	Decellularization of tendons and electrospinning	N/A	Materials used: Rat leg tendons (acellular tendon core) PU and Col I for the middle yarn layer PLLA and BG for the outer nanofiber membrane	Pore morphology: PU/Col I Yarn: The yarn exhibited a parallel nanofiber arrangement, closely resembling the hierarchical structure of natural tendon. The nanofibers in the yarns primarily formed angles ranging from 80° to 90°, which is similar to the collagen fiber arrangement in natural tendons PLLA/BG Nanofiber membrane: The electrospun PLLA/BG nanofiber membrane had a random scaffold structure with equal distribution of nanofibers at all angles. The average diameter of the PLLA/BG nanofibers was reported to be 902.2 ± 172.1 nm	Cell line: TDSCs to evaluate cytocompatibility and the potential of the scaffold to promote tenogenic differentiation, BMSCs to assess the osteogenic potential In vitro results: The decellularized tendons were confirmed to be free of cellular material with retained extracellular macromolecules The PU/Col I yarn showed biomimic properties and promoted the proliferation and TDSCs The PLLA/BG nanofiber membrane, especially at a 2% BG concentration, demonstrated optimal biocompatibility and osteogenic potential, enhancing the differentiation of BMSCs toward osteogenic lineages	Animal model: adult male Sprague Dawley rats, aged 8 weeks and weighing between 250 and 270 g In vivo results: The scaffold was tested in a rat model for anterior cruciate ligament (ACL) reconstruction The composite scaffold showed better integration and mechanical properties compared to controls Micro-CT imaging and biomechanical testing revealed improved bone regeneration and graft—bone integration, especially at 8 weeks post-implantation Histological analyses confirmed the positive effect of scaffold on tendon-to-bone healing, with more mature attachments and expression of tendon-related and bone-related proteins in the complexus group compared to controls	[59]

### Cortical and Cancellous Bone

**Fig. 1:**
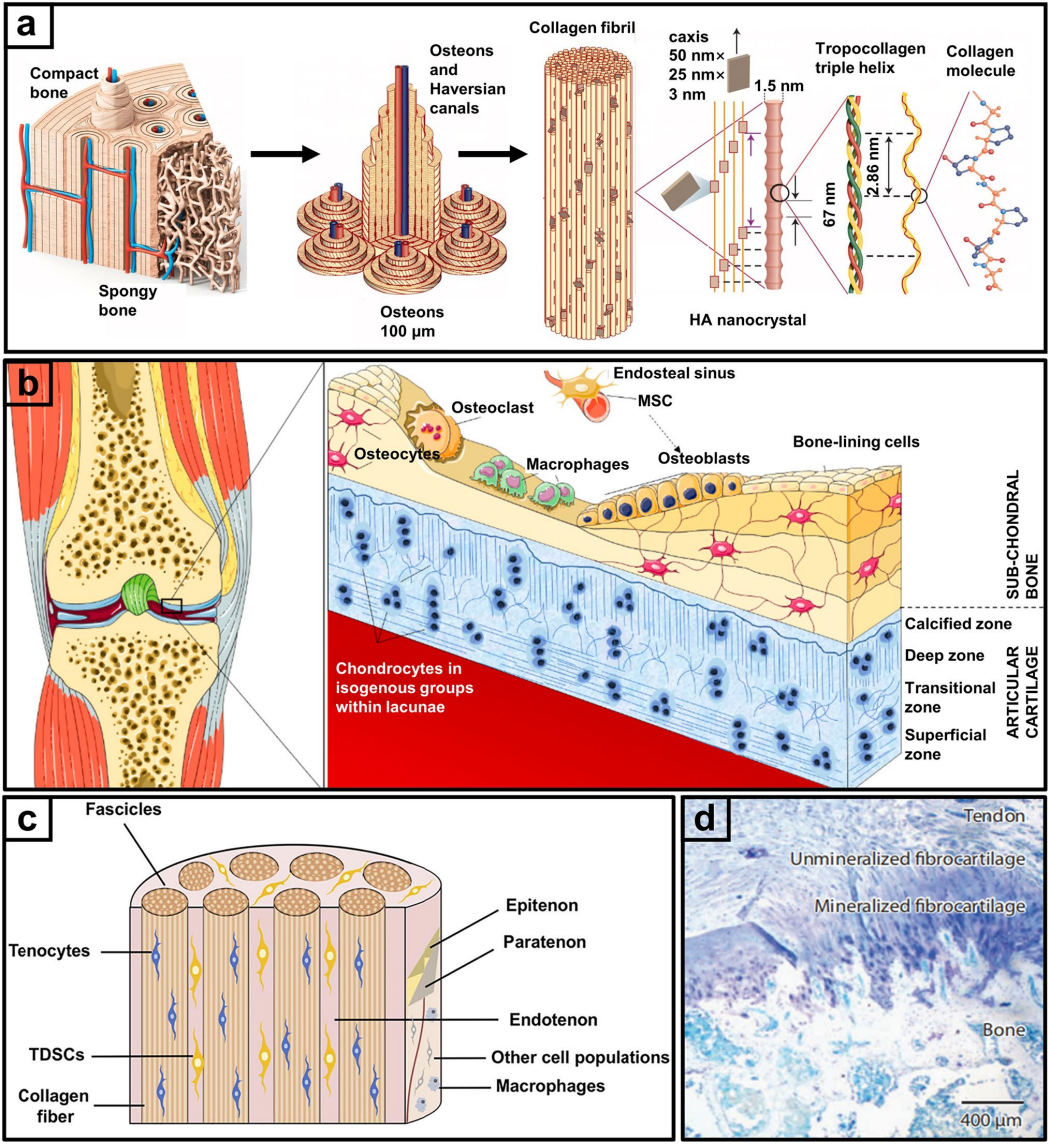
Structure of bone and its interfaces. **a** Bone hierarchical structure [21]. Copyright 2015, Springer Nature. **b** Osteochondral structural view [34]. Copyright 2021, Multidisciplinary Digital Publishing Institute. **c** Microstructure of tendons. (TDSCs: tendon-derived stem cells) [49]. Copyright 2023, Elsevier. **d** The graded hierarchical structure of the tendon-to-bone interface [50]. Copyright 2021, Wiley

Bone is a tough mineralized tissue that provides weight-bearing function to the human skeleton. As shown in Fig. 1a, natural bone contains inorganic components, mainly hydroxyapatite (HAp) crystals, which along with other minerals such as magnesium, sodium, and carbonate ions contribute to the hardness of bone to resist compressive forces. Mineralized bone is responsible for its strength to carry physiological loads. The organic components of bone mainly consist of collagen I (Col I), which allows the bone to withstand bending and tensile forces, and non-collagenous proteins such as osteocalcin and osteonectin, which play a role in mineralization and regulation of bone metabolism [21]. Several cell types maintain bone function including osteoblasts, osteoclasts, and osteocytes. Osteoblasts are mostly located on the bone surface, responsible for forming the bone matrix by secreting organic substances such as collagen protein and inorganic salts [22]. Osteoclasts are present in the internal bone cavities and are responsible for resorbing and remodeling bone tissue. Osteocytes influence the activities of both osteoblasts and osteoclasts, and contribute to the regulation of calcium and phosphate balance [23]. Human long bone comprises the exterior cortical bone, which serves as the primary load-bearing structure, while the interior trabecular bone distributes weight and the marrow cavity transports nutrients [24]. The long bone cross section displays a structural gradient in the radial direction. Cortical bone, also known as compact bone, forms the outer layer of bones and is characterized by a solid and dense structure with a minimal amount of open space. Osteons, also called the Haversian system, are cylindrical structures resembling the fundamental unit of cortical bone [25]. At the center of an osteon is the Haversian canal, a central channel surrounded by a concentric ring of lamellae housing blood vessels and nerves, which provides nutrients and innervation to the bone cells within the osteon. Cancellous bone, referred to as trabecular or spongy bone, exhibits a porous structure suitable for weight distribution. Together, cortical and cancellous bone form a functional gradient structure with distinct composition, porosity and pore size distributions.

Morphologically, the porosity of cortical bone is typically 5%–10%, while that of cancellous bone ranges from 50% to 90% [26]. Pore sizes in cortical bone are relatively smaller compared to cancellous bone, with diameters of 30–50 µm (typically lower than 100 µm). Meanwhile, cancellous bone has larger pores which contribute to its lighter and more flexible nature. The spaces between trabeculae create a network of interconnected pores that vary in size, typically ranging from 300 to 600 µm in diameter. The differences in structure lead to distinct biomechanical properties of cortical and cancellous bone. While cortical bone is strong and hard, making it ideal for weight-bearing and resisting bending or torsional forces, cancellous bone has a trabecular architecture that is not as structurally dense, providing lightweight strength and flexibility. The Young’s modulus of cortical bone is 15–20 GPa, while that of cancellous bone ranges between 0.1 and 2 GPa [27]. Because cortical and cancellous bone acts synergistically to provide the necessary combination of strength, flexibility, and adaptability to the skeleton, their unique functional gradients are a key factor to consider in biomimetic gradient scaffold design.

Designing a scaffold that replicates the cortical-to-cancellous bone gradient is a sophisticated challenge that involves faithfully mimicking the distinct stratification of these two bone types while ensuring a seamless transition between them. This innovative scaffold aims to support the regeneration of both dense cortical bone and porous cancellous bone in cases of bone defects, offering a comprehensive solution for tissue engineering. Critical factors in the manufacturing of these biomimetic scaffolds include achieving a continuous, smooth transition in mechanical stiffness, alongside a progressive increase in pore size and interconnectivity as one moves from the cortical region to the cancellous region [28]. This gradient design is crucial, as it not only meets the mechanical demands of cortical bone but also promotes the biological activities necessary for effective cancellous bone regeneration. Such an approach positions the scaffold as a robust candidate for complex bone tissue engineering applications.

For instance, the study on gradient Voronoi scaffolds demonstrated the application of Ti_6_Al_4_V titanium alloy, showcasing excellent mechanical properties achieved through a controlled gradient design that tailored porosity and pore size distribution [29]. By employing a Voronoi tessellation method, this irregular porous architecture closely resembles the trabecular structure of natural bone. Notably, the gradient Voronoi structure exhibited superior stability and impact resistance compared to regular porous scaffolds, marking it as a promising solution for bone tissue engineering.

Other research studies have explored the use of polymers as substrates, implementing a radial design that mimics the entire cross section of bone [30, 31]. These designs typically feature larger pores at the center, gradually decreasing in size and increasing in density toward the periphery, effectively capturing the essence of natural bone architecture. Moreover, a particular study highlighted the meticulous design of scaffold architecture to replicate the gradient changes found in native bone [32]. In this case, the exterior of the scaffold emulated the dense and robust properties of cortical bone, while the interior transitioned into a more porous structure akin to cancellous bone. This design is not only biomimetic but also strategic, as it provides the necessary mechanical support while allowing for the infiltration of nutrients and cell growth.

### Osteochondral Tissue

Osteochondral tissue is found at the surface of synovial joints, containing stratified regions that form a complex gradient and convey different intra-tissue and inter-tissue functions [33]. Structurally, osteochondral tissue consists of articular cartilage and underlying subchondral bone, while the chondral region can be further divided into continuous zones of superficial, middle, deep, and calcified cartilage (Fig. 1b) [34]. These zones form gradient transition structures that include variations in mineral content, chondrocyte morphology and composition, as well as structural porosity [35–39]. The articular cartilage provides lubrication during repetitive joint motion and distributes loading forces to the underlying hard subchondral bone that provides mechanical support. Due to its low metabolic activity and lack of blood vessels and nerves, cartilage has a limited capacity for self-regeneration that poses substantial challenges to repair following injury [40].

Articular cartilage is composed of chondrocytes embedded in a gel-like ECM, formed from large amounts of collagen and polysaccharides secreted by the chondrocytes [38]. Collagen forms collagen fibers that enhance the strength and toughness of cartilage, while polysaccharides attract and retain water to endow cartilage with its elasticity and resistance to compression [37]. The configuration and organizational pattern of chondrocytes and ECM in different cartilage regions generate a gradient of mechanical properties that is depth dependent, featuring a progressive increase in compressive modulus and strength from the superficial to deep zones [41]. This results from the depth-dependent variation in biochemical composition of osteochondral tissue, whereby collagen content and HAp concentration change from predominantly collagen II and no HAp in the superficial cartilage zone to Col I and abundant HAp in the subchondral bone. Complementing this is the structural gradient of osteochondral tissue with varying porosity, pore size, and pore interconnectivity between layers. The articular cartilage contains mesopores estimated to range in size from 2 to 6 nm, with porosity of 60%–85% and pore sizes gradually increasing from the superficial to the deep zone [11, 42, 43]. This transitions into the underlying subchondral bone, comprising mainly cancellous bone with high porosity from 75 to 90% and large pore sizes of 50 to 300 μm [44]. An ideal osteochondral scaffold should consider a multilayered design with transitional properties to match the structural, mechanical, and biochemical gradients found in native joint tissue.

Osteochondral scaffolds encounter a significant challenge in achieving an optimal balance between mechanical strength and structural integrity. Discrete multilayer scaffolds designed to mimic the unique layers of cartilage, osteochondral interface, and underlying bone often encounter issues such as delamination and mechanical mismatch between different layers. In contrast, scaffolds with smooth mechanical transition designs endeavor to reduce these mismatches by facilitating a gradual transition between softer cartilage and stronger bone. However, achieving this smooth transition is complicated by the significant difference in mechanical stiffness between cartilage and bone tissue[45]. To address this complex dilemma, contemporary research is endeavoring to develop composite materials, graded porosity and innovative hybrid scaffolds.

For example, Zadegan et al. [46] fabricated a three-layer osteochondral scaffold using freeze-drying technology that features a seamless transition between layers . This scaffold integrates silk fibroin (SF) and HA, with the layers having different compositions, resulting in a gradient of mechanical properties. The most striking aspect of this design is the gradient structure, which has been carefully designed to reflect the unique biological and mechanical properties of osteochondral tissue. Clearfield et al. [47] employed a directional freezing method to create a multidirectional scaffold that harnesses both unidirectional freeze casting and lyophilization bonding. This approach successfully replicated the distinct zonal structures of superficial, transitional, calcified cartilage, and osseous zones present in native tissue. The design offered graded pore sizes, anisotropy, and mechanical properties, providing essential cues for directing stem cell differentiation into chondrocytes and osteoblasts.

Golebiowska and Nukavarapu [48] focused on developing bioinspired zonal/gradient scaffolds for osteochondral interface engineering using extrusion-based three dimensional (3D) bioprinting. The study addresses the challenge of replicating the complex hierarchical architecture of the bone–cartilage interface. A key innovation lies in the gradient scaffold architecture, which includes seven zones with gradually changing porosity and infill density to facilitate a smooth transition between the cartilage and bone layers. This structure offers a continuous transition in mechanical properties and pore sizes, ranging from larger pores at the top (for cartilage) to smaller pores at the bottom (for bone). The use of polylactic acid (PLA) as the base material provided sufficient mechanical support, while the integration of cell-laden hydrogel through concurrent bioprinting allowed for selective cellularization of the cartilage zones.

### Tendon-to-Bone Interface

Tendons are mainly composed of densely arranged collagen fibers and tendon cells (Fig. 1c) [49], playing a crucial role in transmitting force and facilitating coordinated movement between muscle and bone. Tendons exhibit gradient transition at the interface with bone (Fig. 1d), which is divided into four regions: tendon, non-mineralized fibrocartilage, mineralized fibrocartilage, and bone [50]. This stratified structure incorporates intricate structural, compositional, and mechanical gradients, along with variations in cellular phenotype and biochemical signals essential for maintaining cell function. The gradient of cellular phenotypes along the tendon-to-bone interface is gradual and continuous, with no clear boundaries between different regions. The tendon region primarily consists of tenocytes, while osteocytes are the main cells found in the bone region [25, 51–53].

Compositionally, the tendon zone contains mostly Col I, while the non-mineralized fibrocartilage contains both collagen II and collagen III with collagen II being more prevalent [54]. The mineralized fibrocartilage contains aggrecan and HAp crystals, along with collagen II and collagen X. The bone zone marks the end of the transitional area, comprising a matrix of mineralized Col I. A variation in collagen fiber orientation also exists, giving the tendon region a denser structure compared to the bone region. Collagen fibers in the tendon zone are aligned in the direction of force transmission, gradually transitioning to a more random and oblique orientation throughout the fibrocartilage zones and eventually becoming interwoven with the mineralized matrix in the bone zone. The most common types of tendon injuries are in the rotator cuff and Achilles, with different mechanical properties and injury patterns that should be considered when designing repair strategies. The ideal gradient scaffolds for regenerating the tendon-to-bone interface should imitate natural tissue transition by incorporating variations in the structure, composition, mechanical properties, and cellular phenotype in a layered or continuous manner to promote functional recovery [55].

Tendon–bone junctions are critical interfaces in the musculoskeletal system, playing a pivotal role in the functional integration of tendons and bones during movement. The current scaffold function on the rotator cuff anatomical site has underscored the importance of developing gradient scaffolds that effectively mimic the natural composition and structure of the tendon–bone interface [56]. These scaffolds are designed to facilitate the seamless transition between the mechanically distinct tissues of tendons and bones, thereby promoting enhanced integration and functional recovery.

For example, a woven scaffold with continuous mineral gradients utilized a combination of electrospinning to create nanofiber yarns with a core sheath structure, paired with traditional textile weaving techniques [57]. This method allows for precise control over fiber orientation and the spatial distribution of mineral content within the scaffold. A novelty of this study is the structural anisotropy, which achieved different mechanical properties in different directions, crucial for replicating the natural anisotropic properties of the tendon-to-bone interface and for providing the appropriate mechanical cues for cell behavior. Another scaffold was designed with a continuous cocktail-like gradient, mimicking the natural transition from tendon to bone [58]. The scaffold comprised a dual-network hydrogel of gelatin methacryloyl (GelMA) and hyaluronic acid, which also incorporated varying concentrations of nanoclay (NC). In addition, the scaffold was loaded with bone marrow mesenchymal stem cells (BMSCs), achieving a smooth gradient transition through a four-layer structure that replicated the natural tendon–bone interface. This scaffold created a gradient of biological signals that promoted osteogenic and tenogenic differentiation while inhibiting adipogenic differentiation, thereby enhancing tendon-to-bone interface regeneration.

Another study applied decellularized tendon as the core of the scaffold, retaining the natural ECM components and tissue strength essential for regulating cell behavior and facilitating in situ tissue regeneration [59]. This design features a complex architecture comprising an acellular tendon core, a middle layer of polyurethane (PU) and collagen I yarn, and an outer layer of poly(L-lactic acid) (PLLA) and bioactive glass (BG) nanofiber membrane. Each layer serves a specific purpose, working in concert to promote effective tissue regeneration and restore the functional integrity of the tendon–bone junction.

The structural features of bone, osteochondral tissue, and tendon-to-bone interface form natural gradients that pose a challenge to recreate using artificial scaffolding strategies. When designing gradient scaffolds to regenerate these musculoskeletal tissues, not only should spatial gradients be incorporated to mimic structural aspects, but also cellular and compositional gradients to maximize tissue repair. Bone repair is a complex process involving inflammation, angiogenesis, soft tissue formation, tissue mineralization, and ultimately bone remodeling to complete long-term healing [2, 21, 60]. The progression of bone repair can be influenced by multiple factors, such as growth factor combinations and concentrations, spatial or temporal delivery of drugs, and selection of repair materials. Meanwhile, the repair of osteochondral and tendon-to-bone interfaces is even more complicated, requiring numerous intricate transitions between materials, pore structure, and biochemical composition, as well as consideration of the potentially conflicting functions of biomolecules in regenerating different tissues [60]. Due to the challenges of using uniform-phase scaffolds in accurately replicating the intricate transitional characteristics inherent to natural tissue interfaces, such as bone–cartilage and bone–tendon, and hence suboptimal physiological and functional restoration outcomes, there is a marked preference toward employing gradient scaffolds to facilitate enhanced repair at musculoskeletal tissue interfaces [61]. Emerging techniques for constructing gradient scaffolds have focused on addressing the challenges of integrating different materials and morphologies to create stratified and connected layers. Meanwhile, other challenges can be encountered in establishing a suitable biochemical gradient for interface tissues because of the overlapping as well as conflicting roles that common musculoskeletal-related growth factors can play in tissue regeneration. For instance, transforming growth factor (TGF)-β and bone morphogenetic protein (BMP)-2 in the regeneration of both cartilage and bone play overlapping/opposite roles [62]. The next section will discuss specific fabrication approaches that have been employed in recent studies to establish biomimetic gradients within biomaterial scaffolds to enhance the regeneration of bone, osteochondral tissue, and the tendon-to-bone interface.

## Manufacturing Techniques for Gradient Bone Scaffolds

The repair of musculoskeletal tissue often involves multiple sites such as bone, articular cartilage, bone-to-cartilage interface, and bone-to-tendon interface. Differences in tissue composition, graded characteristics, and mechanical properties between cortical bone and cancellous bone, bone-to-cartilage, and bone-to-tendon interfaces require unique repair approaches. Various scaffold designs have been adopted to achieve complex tissue regeneration and improve implant integration with host tissues. Fiber membranes are thin structures composed of interconnected fibers, which possess high surface area-to-volume ratio, mechanical strength, and porosity, allowing for efficient nutrient exchange and cell infiltration. They can be fabricated using techniques such as electrospinning, which enables precise control over fiber diameter and alignment. These membranes provide a nanofibrous scaffold for cell attachment, proliferation, and tissue formation, which are a good choice for the regeneration of small-sized bone or tendon injuries without thickness requirements. Hydrogels are 3D networks of crosslinked hydrophilic polymers that can absorb and retain large amounts of water, which closely resemble the ECM and provide a hydrated environment for cell growth. Hydrogels exhibit excellent biocompatibility, tunable mechanical properties, and the ability to encapsulate bioactive molecules. They can be formed through various methods, including physical or chemical crosslinking, and can be designed to mimic the specific properties of the target tissue. In addition, there are also 3D scaffolds composed of polymers, metals, inorganic materials, and their composites prepared by biofabrication techniques, such as fused deposition modeling (FDM) and selective laser melting (SLM). Currently, fibrous scaffolds, hydrogels, and other 3D scaffolds are the mainstream approaches for repairing natural bone, cartilage, tendons, or injuries at interface gradient regions. By selecting different raw material compositions, bioactive substances, and drug concentrations, and combining different scaffold fabrication techniques, it is possible to achieve the desired gradient variation in artificial scaffolds that are compatible with the physiological and structural characteristics of the target natural tissues.

Recent research on repair strategies involving gradient scaffolds has shown promising outcomes in promoting hierarchical tissue healing [63]. As shown in Tables 2, 3, 4, new designs of gradient scaffolds composed of nano-micro materials have been recently enabled by the development of advanced fabrication techniques, such as 3D printing [64, 65], FDM [66], SLM [67], digital light processing (DLP) [68], electrospinning [69, 70], mold-casting hydrogel fabrication [71–73], and microfluidics [74, 75]. These techniques can allow potentially complex, hierarchical gradients to be fabricated in a precise and controlled manner to match the stratified characteristics of native tissues, supplying the biophysical and/or biochemical cues necessary for guiding functional bone, osteochondral, and tendon-to-bone interface regeneration. This section highlights the recent breakthroughs in gradient scaffolds designed to regenerate musculoskeletal tissues, constructed using a variety of fabrication techniques.

**Table 2:** Gradient scaffolds made by electrospinning and other fiber-forming techniques

Application (anatomical site)	Material used/ gradient scaffold architecture/ application	Morphology and mechanical properties	In vitro testing (cell type)	*In-vivo* testing (animal model, species)	Ref
Bone	Material: PCL Poly (methacryloyloxyethyl trimethyl ammonium chloride-co-acrylamide) P(DMC-AMA) Layered gradient scaffold architecture: Upper: a random gelatin fiber structure Middle: dense, highly aligned structure composed of gelatin nanofibers loaded with HA Bottom: dense, highly aligned structure composed of PCL nanofibers loaded with P(DMC-AMA)	Fiber alignment and thickness: The inner face is composed of randomly stacked gelatin (GEL) nanofibers with interconnected 3D porous structures, and the outer face comprises aligned PCL nanofibers GEL fibers-1.9 µm PCL fibers-0.3 µm Mechanical properties: The mechanical strength of Janus guided bone regeneration membrane (JGM) is improved by cross-linking with genipin	Cell line: L929 cells/ MC3T3-E1 In vitro results: The JGM has the potential to be a highly effective scaffold for bone regeneration, combining barrier function, osteogenic stimulation, antibacterial activity, and immune modulation in a single device	Animal model: female New Zealand white rabbits, 12 weeks old *In-vivo* Results: JGM translates its multifunctional properties into living organisms and can promote bone healing, resist bacterial infection, and modulate the immune response	[131]
Tendon–bone interface	Material: SF P(LLA-CL) Layered gradient scaffold architecture: A dual-layer aligned-random nanofibrous scaffold Upper layer with aligned fibers Lower layer with random fibers	Pore size: The average diameter of the aligned layer was 445 ± 180 nm, and the random layer was 486 ± 142 nm Mechanical properties: These small diameters contribute to the high porosity and surface area of the scaffold, which are essential for cell infiltration and tissue ingrowth	N/A	Animal model: New Zealand white rabbits, 6–8 months *In-vivo* Results: The Alizarin Red S (ARS) effectively augmented tendon-to-bone integration and improved the gradient microstructure in the rabbit model by inducing new bone formation, increasing the area of fibrocartilage, and enhancing collagen organization and maturation	[92]
Tendon-bone	Material: PU ICG Continuous gradient scaffold architecture: Gradient in fiber alignment, transitioning from uniaxially aligned fibers to random fibers Gradient in mineral content by controlling the immersion time of the scaffold in a simulated body fluid	Pore size: The average diameter of the nanofibers was 683 ± 215 nm Young's modulus of the mat surface, from 67.78 ± 2.1 MPa (region I) to 4248.6 ± 339.7 MPa (region IV), increased with hierarchical calcium phosphate coating (divided into four regions) Mechanical properties: PU was applied to fabricate electrospun nanofibers, the scaffold was further modified by ICG for photothermal welding	Cell line: TDSCs In vitro results: Scaffolds supported cell attachment and growth and that gradient in fiber alignment and mineral content influenced cell differentiation toward tenogenic (tendon-like) and osteogenic (bone-like) lineages	Animal model: adult New Zealand white rabbits. The injuries of subscapularis tendons *In vivo* Results: Healing was improved in the scaffold group compared to the control group, with increased collagen production and more ordered cellular arrangement at the injury site The scaffolds significantly enhanced the ultimate failure loads of the repaired tendons, indicating improved mechanical strength	[94]
Tendon–bone	Material: Poly-(glycolide-co-caprolactone) (PGCL) SF Mesoporous BGs Layered gradient multilayered structure nanofiber scaffold: Layer 1: SF Layer 2: PGCL/SF Layer 3: PGCL/SF/MBG-0.25 Layer 4: PGCL/SF/MBG-0.5 Layer 5: PGCL/SF/MBG-1 Layer 6: PGCL/SF/MBG-2 Layer 7: PGCL/SF/MBG-4	Pore size: The pore size distributions of SF, SP, SPM, and SPGM were mainly between 2.0 and 50 nm Mechanical properties: The SPGM scaffolds exhibit low stress and high strain; it can deform under load without fracture. The mechanical properties meet the strength requirements for rotator cuff tissue regeneration and repair	Cell line: human skin fibroblasts (HSF-1)/ murine pre-osteoblasts (MC3T3-E1) In vitro results: The SPGM scaffold has promising biocompatibility and the potential to support the growth and differentiation of cells relevant to tendon and bone repair	N/A	[56]
Tendon–bone	Material: PCL Gelatin HA Continuous gradient scaffold architecture: PCL/gelatin fibers to represent the softer tendon tissue PCL/gelatin/HAp fibers to represent the harder bone tissue	Pore size: 1 mL h^−1^ thicker fibers: 368.25 ± 29.47 µm for PCL/gelatin, and 332.99 ± 41.85 µm for PCL/gelatin/HA 0.25 mL h^−1^ thinner fiber: 126.32 ± 7.87 µm for PCL/gelatin, and 182.50 ± 22.22 µm for PCL/gelatin/HA Mechanical properties: PCL/gelatin fibers, the highest extrusion flow rate (1 mL h^-1^) resulted in fibers with lower Young’s modulus 115.30 ± 16.33 MPa, while 0.5 mL h^-1^, 251.80 ± 25.51 MPa; 0.25 mL h^-1^, 241.10 ± 41.92 MPa PCL/gelatin/HAp fibers Young’s modulus ranged from 35.39 ± 6.19 to 59.13 ± 7.87 MPa	Cell line: human adipose-derived stem cells (hASCs) In vitro results: The potential of the wet-spun microfibers to support cell growth, alignment, and differentiation, as well as the ability to induce the formation of an osteogenic-like matrix	N/A	[90]

**Table 3:** Gradient scaffolds made by additive manufacturing and bioprinting

Application (anatomical site)	Material used/ gradient scaffold architecture/ application	Morphology and Mechanical properties	In vitro testing (cell type)	*In-vivo* testing (animal model, species)	Ref
Bone	Material: PLA PCL HAp GelMA DFO Layered gradient scaffold architecture: Structural bi-layered scaffold Biphasic stiff PLA/HAp scaffold with two distinct porous regions	Pore size: PLA/HAp scaffold: outer compact region 481.0 ± 51.7 µm, interior spongy region 887.0 ± 54.4 µm Homogenous GelMA hydrogel: an average pore diameter of 153.02 ± 37.91 µm, distribution in 50–250 µm Mechanical properties: PLA/HAp scaffold: compression modulus 0.90 ± 0.01 GPa, compression strength 58.93 ± 1.46 MPa DFO/PCL/MnCO-GelMA: compression modulus 23.15 ± 1.70 kPa, compression strength 37.22 ± 2.61 kPa	Cell line: MSCs In vitro Results: MSCs are viable and adhere tightly to the scaffold surface The number of MSCs increased significantly with culture time, and the fabricated scaffold exhibited satisfactory cytocompatibility The scaffolds have the potential to promote rapid in-scaffold vessel formation. And scaffolds have a potential function in promoting osteogenic differentiation	Animal model: rat femoral diaphysis *In vivo* Results: The scaffolds are ideally biocompatible and biodegradable *in vivo* over the long term The scaffolds have the potential to promote new regenerative bone formation and accelerate the efficiency of bone repair The scaffolds evoked the formation of a large amount of mature mineralized bone within the defect area	[103]
Bone	Material: PCL GO Layered gradient scaffold architecture: Diverse pore size and porosity Distribution in cortical and cancellous regions	Pore size: Cancellous region: 700 µm (5 × 5 strands pattern) and 900 µm (4 × 4 strands pattern) With increasing GO content in the PCL scaffolds: SEM pore sizes ranged from 721 ± 115 to 998 ± 215 µm, and the experimental porosities ranged from 64.9 to 73.1 Mechanical properties: Young's modulus and compressive strength of the 5 × 5 strands pattern are higher than those of the 4 × 4 strand model, and the increase in GO content decreased the compressive mechanical strength in both dry and wet conditions	Cell line: MG-63 osteoblast cells In vitro results: GO content in the PCL matric accelerated the osteoblasts’ proliferation GO has dose-dependent toxicity. Cell viability did not significantly with the addition of GO	N/A	[26]
Bone	Material: Ti_6_Al_4_V Continuous gradient scaffold architecture: Radial gradient porosity, the pore size within the scaffolds varies radially, with smaller pores near the outer surface and larger pores toward the inner core	Pore size: The average porosity of all as-built samples ranged from 55% to 71%, and the average pore sizes ranged from 333 to 674 µm Mechanical properties: The compression properties results showed that Young's modulus ranged from 2.7 to 7.4 GPa and compressive strength from 255 to 608 MPa. With the addition of the yield strength (233–520 MPa) of all samples, the graded Ti_6_Al_4_V TPMS scaffolds satisfy the requirements of human bone	Cell line: BMSCs In vitro results: Cells adhered to the scaffolds and were densely covered on the surface, with the highest cell adhesion density observed on the T_1.5_ structure, which had the smallest pore size and highest porosity Ti_6_Al_4_V scaffolds with gradient TPMS structures have potential for bone tissue engineering applications, as they support cell growth and show good biocompatibility	N/A	[67]
Bone	Material: β -TCP PCL GelMA Alginate Continuous gradient scaffold architecture: Radial gradient Application: Segmental bone	Pore size: Scaffolds with a 3-iteration fractal-like structure mimic the porosity gradient of natural bone, decreasing from the inner to the outer zone Mechanical properties: The bone-mimicking radial gradient scaffolds with 3-iteration fractal-like structure gradually increases along the radial direction	Cell line: human mesenchymal stem cells (hMSCs) In vitro results: Potential for bioprinting of bone-mimicking radial gradient structures The scaffolds with 3-iteration fractal-like structure have a gradient of gradually decreasing porosity from the inside to the outside, similar to natural bone	N/A	[28]
Osteochondral tissue	Material: PCL Alginate methacrylate (ALMA) Layered gradient scaffold architecture: Tri-layered PCL/ALMA scaffolds Application: Cartilage	Pore size: Superficial layer: filament gap of 300 µm, 0°/90°lay-down pattern Middle layer: filament gap of 500 µm, 0°/60° lay-down pattern Deep layer: filament gap of 700 µm, 0°/30° lay-down pattern Mechanical properties: Superficial layer: tensile modulus 61.57 ± 2.05 MPa and compressive modulus 20.44 ± 1.32 MPa The compressive modulus of the entire PCL/ALMA gradient scaffold was 9.52 ± 1.79 MPa	Cell line: embedded rBMSCs In vitro results: PCL/ALMA hybrid scaffolds exhibited excellent compatibility with rBMSCs ALMA furnished an appropriate cartilage growth microenvironment, with evidence of some larger cell masses and homogeneous distribution There was greater upregulation of chondrogenic-specific gene expression	N/A	[45]
Osteochondral tissue	Material: PCL MMP Diclofenac sodium (DC) KGN b-TCP BMSCs Layered gradient scaffold architecture: Top layer DC-loaded MMP Middle layer for cartilage regeneration: a double network of BMSC-laden HAMA hydrogels and PCL (KGN) Bottom layer for subchondral bone regeneration: PCL (b-TCP) porous scaffold Application: Osteochondral	Pore size: PCL (β-TCP) scaffold: elastic modulus ~ 580 kPa, stiffness ~ 350 N mm^-1^, and ultimate stress ~ 460 MPa Mechanical properties: Read from the graphs	Cell line: rBMSCs were incorporated into HAMA Bioink: BMSCs-laden HAMA In vitro results: The proliferation of BMSCs was significantly enhanced compared to the control group DC-loaded composite scaffold significantly decreased IL-1β levels by 46.4% compared to the control The scaffolds significantly inhibited IL-1β expression in BMSCs	Animal model: female Lewis rats, 6-week-old In vivo results: MMP-HAMA hydrogels have been observed to enhance chondrogenesis, promote cartilage matrix deposition, inhibit MSC hypertrophy, and reduce cellular calcification BMSC-laden scaffold treatments reveal potential chondroprotection and anti-inflammatory effects BMSC-laden bioprinting facilitates improved recovery of joint function, strength and walking pattern/speed after joint injury The β-TCP and porous scaffold structure in the designed scaffold stimulated the regeneration of subchondral bone	[106]
Osteochondral tissue	Material: SF Parathyroid Hormone (PTH) GelMA Articular chondrocytes (ACs) BMSCs Layered gradient scaffold architecture: Structural biphasic scaffold with a mechanical gradient on the basis of dual modification of SF Articular cartilage: GelMA + SF-PTH (added ACs) Subchondral bone: GelMA + SF-MA (added BMSCs) Application: Osteochondral	Pore size: Approximately 150–300 µm Mechanical properties: 10% GelMA-5% SF-PTH inks (Cartilage layer) have a compressive strength of 180 kPa 10% GelMA-5% SF-MA inks (bone layer) have a compressive strength of 260 kPa	Cell line: rabbit ACs / BMSCs In vitro results: GelMA + SF-MA bioink had good mechanical properties GelMA + SF-PTH bioink inhibited chondrocyte hypertrophy and favored hyaline cartilage ECM production	Animal model: adult male New Zealand white rabbits In vivo Results: GelMA + SF-PTH/GelMA + SF-MA scaffolds can promote the regeneration of osteochondral defects and maintain the hyaline cartilage phenotype to a large extent	[132]

**Table 4:** Gradient scaffolds made by sequential layering of hydrogels

Application (anatomical site)	Material used/ gradient scaffold architecture/ application	Morphology and Mechanical properties	In vitro testing (cell type)	*In-vivo* testing (animal model, species)	Ref
Osteochondral tissue	Material: PCEC GelMA PLGA BMSCs BMP-2 TGF-b1 BMP-7 Layered gradient scaffold architecture: S: 6 mg BMP-7@PLGA /mL + 2 mg TGF-β1@PLGA /mL D: 8 mg TGF-β1@PLGA /mL B: 10 mg BMP-2@PLGA /mL Application: Osteochondral	Pore size: Fiber diameters, printed via MEW with different fiber spacings: 100 μm for the superficial cartilage (S) layer, 200 μm for the deep cartilage (D) layer and 600 μm for the subchondral bone (B) layer; lay-down patterns (0◦-30◦ for the S layer and 0◦-90◦ for the D and B layers) Scaffold porosities: S layer 86.89%, D layer 92.27% and B layer 60.46% Thickness of in vivo implanted scaffold: S layer 150 μm, D layer450 μm, and B layer 2.5 mm Spherical PLGA microspheres with an average diameter of 3.12 ± 0.87 μm Compressive mechanical properties (*E*): The compressive modulus for GelMA hydrogel: 28.4 ± 2.3 kPa Fibrous networks alone for the S layer 46.1 ± 3.8 kPa, D layer 34.8 ± 2.7 kPa, and for the SD layer 165.8 ± 27.6 kPa *E* is increased in the S layer-reinforced construct (283.6 ± 22.3 kPa), in the D layer-reinforced construct (256.6 ± 24.9 kPa), and in the SD layer reinforcement 964.2 ± 56.8 kPa * E* of the bone (B) layer: 46.1 ± 4.2 MPa for the scaffold alone, and 55.8 ± 5.4 MPa for the PCEC/hydrogel composite	Cell culture: Chondrogenic and Osteogenic In vitro results: The infilled hydrogels and PLGA microspheres did not negatively affect the cell viability The results reflected the stratified osteochondral tissue function in guiding native-like cell orientation and zonal marker protein deposition	Animal model: New Zealand white rabbits, three-month-old In-vivo Results: Fiber-reinforced tri-layered constructs can provide lubrication resembling the native cartilage surface and the stratified structure enables the entire osteochondral tissue repair	[89]
Osteochondral tissue	Material: GelMA Alginate Methacryloyl BMSCs Layered gradient scaffold architecture: Bilayer and cell-specific stratified structure Upper superficial chondroid ECM-loaded chondrocytes Lower deep osteoid ECM-loaded BMSCs Application: Osteochondral	N/A	Cell culture: articular cartilage progenitor cells (ACPCs)/ BMSCs In vitro results: BMSCs showed excellent adipogenic and osteogenic differentiation, whereas ACPCs exhibited enhanced chondrogenic properties The scaffold achieved ACPC and BMSC commitment toward chondrocytes or osteoblasts in vitro 3D culture	Animal model: rabbits, osteochondral defect modeled in the femoral trochlear groove In vivo results: Both BMSC- and ACPC-laden hydrogels reproduced a new osteochondral unit The scaffold can promote bone phase regeneration and maturation	[144]
Osteochondral tissue	Material: GelMA SAMA b-TCP KGN BMSCs SA Layered gradient composition, structural biphasic hydrogel scaffold: Chondral layer hydrogel (CLH) SAMA/GelMA (2 wt%:3 wt%, with a high concentration of KGN) Osseous layer hydrogel (OLH) SAMA/GelMA/β-TCP (1 wt%:1 wt%: 0.5 wt% β-TCP) and low concentration of KGN Application: Osteochondral	Pore sizes: CLH: 150–200 µm OLH: 200–300 µm Mechanical properties: Final compressive strength of the biphasic CLH/OLH scaffold: 0.24 ± 0.03 Mpa	Cell culture: BMSCs In vitro results: The addition of β-TCP showed a promotion for the cell proliferation Hydrogel with the incorporation of KGN exhibited a higher differentiation of chondrocytes Synergic effect of β-TCP and KGN on the osteogenic differentiation of BMSCs in OLH, the secretion of COL I and COL X was more in the osseous layer with β-TCP than in the chondral layer	Animal model: rats Knee joint trochlear site, a cylindrical defect (2.0 mm in diameter, 1.6 mm in depth) In vivo Results: Synergistic utilization of KGN with β-TCP in the osseous layer can provide a good microenvironment for osteochondral repair	[114]
Osteochondral tissue	Material: COL I HAp Continuous compositional gradient scaffold: Cartilage-like region: HAp/COL I 0/100 Upper middle zone: HAp/COL I 10/90 Lower middle zone: HAp/COL I 30/70 Bone-like region: HAp/COL I 50/50	Pore sizes: HAp content did not significantly change the pore shape and distribution; however, it increased the roughness of the pore wall surfaces Mechanical properties: The entire gradient scaffold displayed a compressive modulus of about 15 kPa The depth-dependent gradient stiffness of the scaffold was determined by the HAp content	Cell culture: BMSCs In vitro results: Regions with a higher content of chondrogenic collagen and relatively lower stiffness substrate favored cell proliferation in chondrogenic condition Higher HAp content and resulted in higher stiffness favored cell activity in osteogenic condition	Animal model: female Fischer 344 rats, 12 weeks old In vivo Results: The gradient scaffold was colonized by host cells after 15 days, highly stromal fibrous tissue Mineral inflammatory response, the scaffold was biocompatible	[116]
Tendon–bone	Material: Gelatin Cu^2+^ Zn^2+^ Layered gradient scaffold architecture: Gradient bimetallic (Cu and Zn) ion-based hydrogel Application: Rotator cuff	SEM: ion-based hydrogels, uniform porous micromorphology Mechanical properties: The release profiles of Cu^2+^ and Zn^2+^ were analyzed using inductively coupled plasma mass spectrometry, which showed an initial rapid release followed by a continuous slow release	Cell culture: rat MSCs In vitro results: The ion-based hydrogels showed clear antibacterial activity The hydrogel did not have a negative impact on cell morphology The hydrogel could promote the osteogenic differentiation of osteoblasts and the proliferation of tenocytes	Animal model: Sprague–Dawley rat, the rat rotator cuff tear model In vivo results: The gradient bimetallic ion-based hydrogel has significant potential as a biomimetic material for promoting the repair and regeneration of complex tissues	[120]
Tendon–bone	Material: PCL Calcium phosphate silicate (CPS) Layered gradient scaffold architecture: A series of layer with different P/C ratios and form the gradient film Application: Tendon-to-bone	N/A	Cell line: Osteoblasts In vitro results: As the CPS content on the composite film increased, the cells showed increased adhesion and better growth Osteoblasts on layers with higher CPS content induce mineralization more efficiently	Animal model: New Zealand white male rabbits, 5 months In vivo results: The G-P/C composite film can gradually augment osseointegration of the tendon and bone, improve biomechanical properties, and promote a more natural healing process at the tendon-to-bone interface The gradient structure and composition of the film appear to positively influence the healing process	[129]

### Gradient Scaffolds Made by Electrospinning and Other Fiber-Forming Techniques

The ECM of most musculoskeletal tissues comprises an intricate structure of collagen fibers, which directly interacts with cells and serves as an active reservoir for regulating growth factor activity. A primary aim of engineering musculoskeletal tissues is to mimic the ECM structure using micro- and nanofibrous materials, prepared using a variety of methods such as self-assembly [76], phase separation [77], wet spinning, and electrospinning [78]. Among these, electrospinning has been widely adopted in tissue engineering for producing nano-sized fibers or fibrous membranes with large surface area-to-volume ratio and high porosity, which may imitate the collagen fiber arrangements found in bone and related interfacial tissues [79]. Electrospinning uses the electrostatic repulsive force generated from differences in surface charge to eject nanofibers from a viscoelastic fluid [80]. As shown in Table 2, it has been a favored technique for constructing anisotropic or gradient scaffolds for musculoskeletal tissue engineering due to its flexibility in processing various materials (including organic, inorganic, and composite materials), adjusting a range of material properties (including diameter, porosity, and thickness), and realizing customized scaffold designs (such as aligned, hollow, and core sheath).

**Fig. 2:**
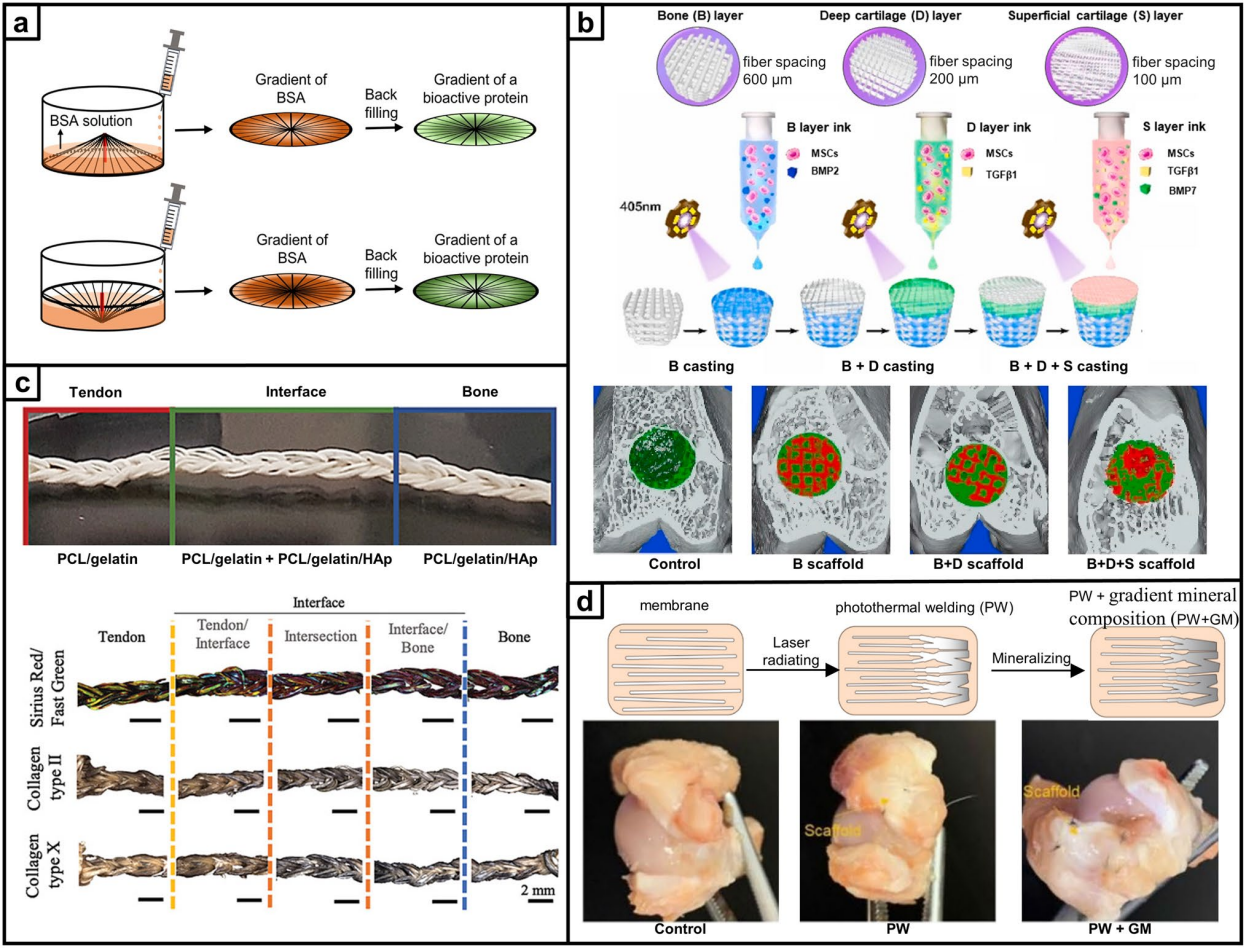
Electrospinning for preparing gradient biomimetic scaffolds. **a** The preparation procedure of gradient protein on the electrospun fiber scaffolds [88]. Copyright 2018, American Chemical Society. **b** The tri-layer scaffold for osteochondral repair constructed using melt electrowriting and UV-assisted stepwise infiltration and cross-linking and the transverse view of the 3D reconstruction images of bone repair at 24 weeks post-surgery for the different groups (The off-white color, green color and red color in 3D reconstruction images represent the primary bone, the regenerated bone and the implanted scaffold, respectively) [89]. Copyright 2020, Elsevier. **c** A woven scaffold for biomimetic tendon repair formed by wet electrospinning with a gradient of HAp and Sirius Red/Fast Green staining and DAB-immunostaining exposure revealed a mineralization gradient of collagen II and collagen X [90]. Copyright 2019, Wiley. **d** A dual-gradient electrospun scaffold prepared using photothermal welding and gradient mineral deposition for rotator cuff injury repair and the photographs of specimens retrieved at six weeks post-operation. [94]. Copyright 2022, Springer Nature

Electrospun nanofibrous scaffolds with a variety of properties have been tested for their reparative effects in bone regeneration, including those with different sizes, structures, composition, morphology, porosity, and assembly [81]. The arrangement of fibers can be controlled by adjusting the electrospinning parameters, resulting in aligned or random structures. Different fiber structures also exhibit variations in porosity and morphology, which may influence cell behavior. For example, aligned and random nanofibrous membranes prepared from the same material were found to affect the behavior of BMSCs, whereby cells migrated along the direction of aligned fibers but exhibited random and disordered migration on random fibers [82]. Therefore, the biomimetic structural characteristics of electrospun nanofibers play a crucial role in promoting cell growth and guiding tissue regeneration [83, 84]. Various methods are therefore used to construct electrospun nanofibrous membranes with oriented arrangements to confer tissue mimetic characteristics, such as structural gradients in poly(lactic-co-glycolic) acid (PLGA) nanofibrous membranes comprising graded arrangements and porosities, constructed by adjusting the solvent exposure [85]. Assisted by the introduction of magnetic poles, electrospun fibers can also be made to gradually transition from being highly aligned in the presence of the magnetic field to being randomly aligned away from the magnetic field, to mimic the structural gradients found in native tissues [86]. In addition to biophysical guidance conferred by nanofibrous materials, biochemical gradients with variations in the density of bioactive substances play a significant complementary role in directing cell behavior. For example, protein gradients have emerged as a powerful means of enhancing tissue regeneration by directing cell migration, extension, and differentiation. Combining specifically designed protein gradients with scaffolds made from aligned polymer fibers can significantly improve tissue regeneration outcomes by further accelerating cell proliferation and migration [87]. The formation of a protein gradient on the fiber membrane can be achieved by multi-step immersion of the membrane in protein solution, which may be cumbersome, or by masking the membrane with a gradient of ‘mask’ protein (such as bovine serum albumin (BSA)), which forms a gradient by controlling the BSA concentration or deposition time (Fig. 2a) [88]. The bioactive protein of interest is then used to fill the gaps on the membrane that are not blocked by BSA, resulting in a functional gradient that may help direct anisotropic tissue regeneration.

To construct interfacial scaffold regions, the incorporation of specific types or varying concentrations of bioactive substances is strategically implemented, resulting in biochemically layered characteristics that may facilitate anisotropic tissue repair. For example, by combining melt electrospinning for microfiber fabrication and FDM of biomaterials, melt electrowriting (MEW) that benefits both technologies can be maximized to create a scaffold that replicates the intricate structure and function of native osteochondral tissue. In one study, a tri-layered fiber hydrogel scaffold was constructed by MEW from triblock polymer of poly (*ε*-caprolactone) (PCL) and poly(ethylene glycol) (PCEC) networks with depth-dependent fiber organization [89]. GelMA hydrogel loaded with marrow mesenchymal stem cells (MSCs) and growth factors in different regions in the fiber hydrogel scaffold exhibited the capability for zone-specific delivery of growth factors, as shown in Fig. 2b. By varying the fiber configuration and material composition gradient, this bioinspired scaffold aimed to induce region-specific cartilage and bone differentiation to restore functionally stratified osteochondral tissue. In vivo experiments, rabbit osteochondral defect models demonstrated that the three-layer scaffold could enhance the wear resistance and lubrication qualities of newly formed osteochondral tissue, significantly improving the regeneration of both cartilage and subchondral bone.

Given the specific voltage and temperature requirements of electrospinning, there is a risk of denaturing the structure of natural polymer materials. For this reason, traditional electrospinning is commonly applied to synthetic polymers which lack bioactivity. Wet spinning is a method for manufacturing polymer fibers, where the polymer solution can be extruded into a supportive solidification bath to prepare fibers without the use of high temperatures or pressures. To construct fibrous scaffolds for tendon-to-bone healing that incorporate natural polymers, wet spinning has been employed to manufacture continuous composite microfibers targeted at the hierarchical transition region between tendon and bone. In one study, two different types of wet-spun microfibers were produced: PCL/gelatin and PCL/gelatin/HA, whereby the microfiber composition and structure were altered to, respectively, replicate the anisotropic arrangement of tendon and mineral content of bone [90]. Uniquely, the scaffold was constructed through textile assembling of microfibers by knitting, creating 3D fibrous structures with continuous topographical and compositional gradients to mimic the native tendon-to-bone transition. The topological structure and compositional variances within gradient scaffolds influenced the differential deposition of collagen proteins across distinct structural regions. Specifically, staining results revealed heightened levels of non-collagenous proteins within the tendon segment, while the interface region exhibited notably increased concentrations of collagen II and collagen X. This collagen deposition profile mirrored the structure of native tendon tissue, confirming the ability of the scaffold to promote tissue regeneration replicating natural ECM distribution patterns (Fig. 2c). Wet-spinning reduces denaturation and inactivation of biomaterials due to mild production conditions. However, organic solvents are still required to formulate the spinning liquid, and non-environmentally friendly coagulation baths are sometimes used. In addition, it is difficult to synthesize fibers with nanoscale diameter using this method, which may limit its ability to produce scaffolds that regulate bone-related tissue regeneration on microscopic levels.

Biomimetic structural gradients play a pivotal role in interface tissue repair due to their ability to effectively mitigate scar formation during tissue healing processes [91]. To mimic the gradient structure of natural tendons, a SF/poly(l-lactic acid-co-caprolactone) (SF/P(LLA-CL)) nanofibrous scaffold was constructed by electrospinning with a syringe pump [92]. A dual-layer aligned-random nanofibrous scaffold was created, where the upper layer consisted of aligned fibers with diameters of 445 ± 180 nm and the lower layer consisted of randomly distributed fibers with diameters of 486 ± 142 nm. When used to repair Achilles tendon injuries in New Zealand white rabbits, the gradient nanofibrous scaffolds significantly enhanced tendon-to-bone healing compared to scaffolds with random fibers only, evidenced by improved mechanical properties and bone regeneration at the interface region. The functionalization of scaffolds with both fiber alignment and a gradient of mineral content can confer more remarkable repair effects compared to individual strategies [93]. In another study, a photothermal welding technique was applied to an electrospun scaffold to establish a gradient of fiber alignment, which was then modified with a graded mineralization coating to mimic the natural tendon–bone interface, as shown in Fig. 2d [94]. PU/indocyanine green (PU/ICG) nanofiber scaffolds were created using electrospinning, with ICG acting as a photothermal agent. Exposure to near-infrared laser caused the fibers to weld at cross-points due to heat generated by ICG, allowing for controlled fiber alignment from uniaxial to random orientations by adjusting laser irradiation time and intensity. The scaffolds, immersed in simulated body fluid for varying durations, developed a dual-gradient structure with increasing mineral deposition over time and decreasing fiber alignment. This scaffold mimicked the natural tendon-to-bone interface, supported cell growth across all regions, and histological images taken at six weeks post-operation revealed nearly complete healing of rabbit rotator cuff injury with no scar formation in the dual-gradient scaffold group, in contrast with poor healing in the control and single-gradient scaffold groups. These findings suggest that multi-gradient biomimetic scaffolds resembling natural tissues might be more effective at promoting the repair of interface tissues.

Nanofibrous scaffolds, including those prepared using electrospinning, are typically made as fibrous membranes exhibiting a thickened 2D structure, which may be difficult to satisfy the thickness requirements of certain musculoskeletal tissue structures. It is challenging to create structurally intricate scaffolds solely through traditional electrospinning. To address this problem, 3D fiber scaffolds with gradient structure can be generated by combining electrospinning with foaming method or by assembling short fibers obtained by mechanical cutting of continuous fibers down to the micron level [95]. Typically, a hybrid fabrication approach combining multiple methods is necessary to realize complex gradient scaffold designs and circumvent the limitations of individual techniques. Future strategies would benefit from the simultaneous generation of structural and compositional gradients within scaffolds to promote optimal healing at musculoskeletal tissue interfaces.

### Gradient Scaffolds Made by Additive Manufacturing

Compared to scaffolds built up from 2D fibrous membranes, scaffolds with a 3D structure may be more beneficial for tissue repair, particularly for tissues exceeding a few millimeters in depth, by facilitating better infiltration and growth of cells [51]. Various methods can be used for preparing 3D scaffolds, such as gas foaming [96], dispersion shaping [97], sacrificial components, and additive manufacturing [65, 98]. Among these, additive manufacturing including technologies such as 3D printing and bioprinting is becoming increasingly popular due to its precision and capacity to allow customization compared to conventional, more manual fabrication techniques. Additive manufacturing allows the fabrication of complex 3D structures layer by layer, enabling the precise control of scaffold architecture, porosity, and mechanical properties. Intricate and hierarchical scaffold designs with highly precise internal and external geometry can be realized through the controlled deposition of material building blocks, which may be spatially tailored to the requirements of the target tissue. Moreover, complex scaffold shapes and geometries can be fabricated to create patient-specific scaffold implants that can accommodate individual variations in the anatomy or structure of the target tissue or organ. It incorporates multiple materials with different properties into a single scaffold, which closely mimics the structure and function of native tissue while promoting tissue integration and regeneration. Current additive manufacturing supports a wide range of materials selection, including natural and synthetic polymers, hydrogels, bioceramics, and composites. As shown in Table 3, variations in material composition and pore structure within the scaffold can be precisely realized with additive manufacturing, for instance, through layer-by-layer printing to create stratified structures suitable for the regeneration of gradient tissues. These scaffolds are expected to resemble the natural tissue environment, with the necessary mechanical properties and bioactive functions to promote cell attachment, nutrient diffusion, and tissue regeneration [99].

Gradients in mineral content, cellular composition, and structural porosity form important features in cortical and cancellous bone. Additive manufacturing of gradient scaffolds for bone repair often presents a multilayered design that includes anisotropic pore structures with varying pore diameters, shapes, spacing, and arrangements. Garg et al. explored how pore and fiber sizes in electrospun scaffolds affect macrophage polarization in vitro, revealing that larger fibers and pore sizes promote macrophage polarization toward a regenerative M2 phenotype [100]. Conversely, another study showed that gelatin scaffolds formed by cryogelation with 30 μm pore size favored the M2 phenotype, while 80 μm pore size induced the M1 phenotype [101]. The optimal pore size in scaffolds for musculoskeletal tissue healing remains debated, and a definitive conclusion of its impact on macrophage polarization has yet to be reached. Combined with variations in material composition and mechanical properties, these gradient scaffolds can help induce patterns of cell differentiation replicating the processes necessary for the formation of bone and related tissues [102]. In addition, as bone repair involves different stages of healing, the repair outcomes may be enhanced by supplementing the 3D scaffold with concentration gradients of various bioactive substances.

**Fig. 3:**
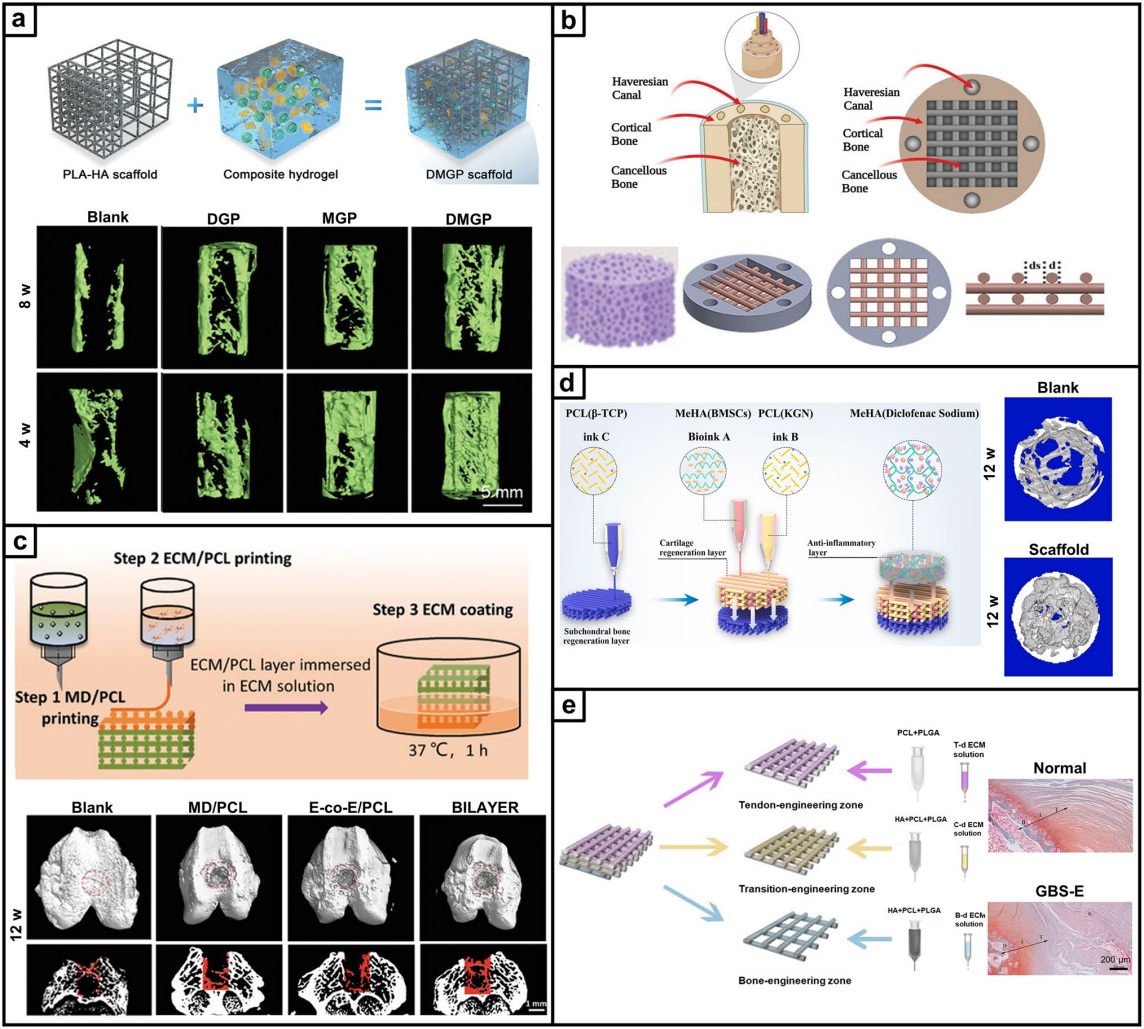
Gradient scaffolds fabricated by 3D printing. **a** Schematic of a biomimetically hierarchical scaffold designed to enhance bone regeneration and micro-CT images of 3D reconstruction showing regenerated bone around the defect in the untreated blank group and three other experimental groups at 4 and 8 weeks post-surgery. (DGP:DFO@PCL-GelMA-PLA-HA, MGP: MnCO-GelMA-PLA-HA, DMGP: DFO@PCL-MnCO-GelMA-PLA-HA) [103]. Copyright 2022, Wiley. **b** Gradient scaffold design for regeneration of cortical and trabecular bone [26]. Copyright 2022, Wiley. **c** Scheme of the preparation process of a bilayer scaffold for osteochondral repair and the 3D reconstruction images of the subchondral bone regenerated in the defects in different groups at 12 weeks after surgery (MD/PCL: PCL-based scaffold incorporating MgO@PDA, ECM/PCL:ECM-incorporated PCL-based 3D printed scaffold, E-co-E/PCL: ECM/PCL coated with ECM hydrogel) [105]. Copyright 2023, Wiley. **d** A tri-layer scaffold for osteochondral repair made using 3D printing technology combined with hydrogel and 3D reconstructed micro-CT images of the osteochondral defect areas in the blank group and scaffold group at 12 weeks post-surgery [106]. Copyright 2021, Elsevier. **e** A biomimetic tri-layer scaffold with gradient composition and structure for rotator cuff repair and safranin O staining of the repaired tendon-to-bone site at 16 weeks postoperatively. (GBS-E: mechanics-graded biomimetic scaffold with decellularized ECM) [107]. Copyright 2023, American Chemical Society

In one study, a two-layered PLA-HAp scaffold with a biomimetic gradient of pore sizes was fabricated by FDM to replicate the structure of cortical and cancellous bone, as shown in Fig. 3a [103]. The pore sizes varied from 430 μm in the outer cortical region to 900 μm in the inner cancellous region. Pore sizes in the range of 250–500 μm are favorable for ECM secretion, while large pore sizes above 500 μm stimulate the growth of vascular tissues, thereby accelerating the bone repair process. To endow the hard scaffold with ECM-like properties, the base scaffold was injected with a GelMA-based soft hydrogel encapsulating deferoxamine@PCL (DFO@PCL) nanoparticles and manganese carbonyl (MnCO) nanosheets for suppressing inflammatory response and promoting angiogenesis. DFO@PCL nanoparticles showed an initial burst drug release of 22.67 ± 0.68% at 1 day, followed by sustained slow release of approximately 45% of the drug at 13 days. DFO inhibited osteoclast differentiation by suppressing the electron transport chain and negatively regulating the activation of mitogen-activated protein kinases [104], synergistically acting with the osteogenic properties of the base scaffold to provide ‘osteoimmunomodulation’ function and leading to enhanced bone formation. This hybrid scaffold showed significant ability to induce in vitro osteogenic gene expression by MSCs and downregulation of inflammatory mediators in macrophages. The anti-inflammatory effects were the result of continuous release of CO and Mn^2+^ from the scaffold, while the interaction of DFO and MnCO was thought to drive angiogenic processes. After implanting the scaffold in a critically sized femoral defect in rats, micro-CT imaging showed that compared with other groups, the defect area was almost completely healed in the osteoimmunity-regulating scaffold group, which had the highest new bone formation rate (25.74% ± 2.96%). By providing multi-dimensional biomimicry of natural tissues in structure, composition, and the biological processes of repair, this scaffold was considered a candidate for promoting large-scale repair of bone defects.

The regeneration of structurally biomimetic cortical bone has been a longstanding challenge, as it comprises a dense layer of exterior tissue harboring interior Haversian canals with microscopic tubes or tunnels. The Haversian canals also contain nerve fibers, blood vessels, and lymphatic vessels to allow communication between osteocytes and nutrient transport [25]. To replicate this complex structure, a scaffold with radially gradient pores was fabricated using FDM technology [26]. The scaffold comprised a cortical region to mimic the Haversian channels and a cancellous region with interconnected lattice structures. The outer cortical region presented a densified radial structure mimicking the cross section of long bone, with four holes of approximately 1200 μm diameter resembling Haversian channels, while the large inner cancellous region contained trabecular beams consisting of interconnected lattice strand patterns. PCL was used as the primary scaffold material due to its good printability, incorporated with graphene oxide (GO) nanoparticles at two different concentrations (0.25% and 0.75% w/w) to enhance hydrophilicity and mechanical properties (Fig. 3b). The scaffold showed elastic modulus matching the ranges of values for cancellous bone with acceptable biocompatibility, although its ability to induce bone regeneration remains to be verified in vivo*.*

Although multilayered or gradient scaffolds can better satisfy the regenerative requirements of different tissue types for interfacial tissue regeneration, delamination between scaffold layers poses a common challenge. Additive manufacturing provides a convenient preparation method for integrated scaffolds featuring regions with different properties while avoiding problems with separation between layers. For example, low-temperature deposition manufacturing (LDM) was applied to generate bilayered scaffolds through different modifications to PCL as the base material in both layers for osteochondral tissue repair [105]. As shown in Fig. 3c, the upper cartilage layer comprised PCL incorporated with porcine cartilage ECM and further coated with ECM hydrogel, while the bottom bone layer comprised magnesium oxide nanoparticles (MgO) modified with polydopamine (MgO@PDA). Coating the ECM hydrogel improved not only the cell affinity but also the interfacial force between the scaffold layers. The tensile fracture energy of composite scaffolds at the interface was not significantly different from that of pure PCL scaffolds. The presence of ECM in the cartilage layer was conducive to chondrocyte adhesion and migration, endowing it with chondrogenic potential, while the ability to release Mg^2+^ in the bone layer conferred an osteopromotive effect that was beneficial in early osteogenesis. The expression of osteogenic differentiation-related genes in the composite scaffold group was approximately two times that of the PCL scaffold, as was the cell proliferation profile. In vivo implantation of this scaffold in a rat osteochondral defect model resulted in complete regeneration of the cartilage tidemarks after 12 weeks, with no tissue separation between the cartilage and bone layers. The rate of new bone production and bone density in the group with composite scaffolds was 1.4 times higher than in the control group.

Cellular components can be further integrated into gradient scaffolds by bioplotting. For example, bioplotting BMSC-laden scaffolds were developed for treating osteochondral defects associated with osteoarthritis, combining cartilage regeneration with inflammation management [106]. The bioprinted gradient scaffolds consisted of three distinct layers, each with a specific function (Fig. 3d). The bottom layer was a porous scaffold comprising PCL and β-tricalcium phosphate (β-TCP) to mimic the structure of subchondral bone. The middle layer was PCL loaded with kartogenin (KGN) and methacrylated hyaluronic acid (HAMA) together with BMSCs for cartilage regeneration, printed in an alternating pattern. The top layer was a coating of HAMA hydrogel encapsulating diclofenac sodium as an anti-inflammatory agent that was sensitive to matrix metalloproteinase (MMP) cleaving for release. The multiple functions of the scaffold in facilitating simultaneous osteogenesis, chondrogenesis, and suppression of inflammation were found to be effective in repairing osteochondral defects in a rat model of injury-induced osteoarthritis. The scaffold-implanted joints showed significantly improved joint function and inhibited the worsening progression of osteoarthritis through cartilage regeneration. The 12-week micro-CT imaging results from in vivo implantation indicated that the use of gradient scaffolding significantly enhanced the regeneration of new bone. This study suggested that time-dependent release of bioactive substances along a gradient scaffold to match different stages of the repair process can be a promising strategy in musculoskeletal tissue engineering, particularly for repairing interface tissues.

To achieve tendon-to-bone repair, bioplotting was applied to generate a unique scaffold containing three different types of gradients: structure, composition, and mechanics, to mimic the native tendon, fibrocartilage, and bone regions (Fig. 3e) [107]. A material gradient was constructed using combinations of PCL, PLGA, and HAp in different proportions to mimic the transition in composition and mechanics within tendon–bone tissue, with increasing HAp content from the top to bottom layer. This was complemented with an increase in pore sizes from 150 μm in the densest tendon region, to 150–250 μm in the intermediate fibrocartilage region, and up to 300–400 μm in the bone region. The optimal pore sizes for tenogenic, chondrogenic, and osteogenesis differentiation were 150, 150–250, and 300–400 μm, respectively. 150 μm pore size was selected for tendon and transition zones to favor inward cell growth, while 300 μm pore size was suitable for skeletal zones. Moreover, the entire scaffold was coated with decellularized tendon, cartilage, and bone ECM from rabbits in the respective regions to enhance tissue-specific structure and bioactive properties. When applied to a rabbit model of rotator cuff injury, this triple gradient scaffold was able to restore a tendon-to-bone interface resembling native tissue transition after 16 weeks, together with significantly improved biomechanical properties.

The successes obtained thus far with additive manufacturing to fabricate scaffolds for the regeneration of musculoskeletal tissues have mostly taken advantage of the ability of these technologies to offer customization and precise control. Using computer-aided design, additive manufacturing allows gradient structures to be generated that precisely match irregular defects or complex scaffold geometries. Achieving continuous compositional gradients allows for better tissue simulation. Nevertheless, most current scaffolds only change composition or structure to achieve discrete gradients. Transitions in properties within the scaffold are stepped and discrete, not continuous. Mixed control of parameters such as material composition and structure through multi-system fabrication methods, or hybrid additive manufacturing platforms with multiple nozzles are noteworthy development directions for creating scaffolds with continuous gradients [108, 109].

In addition, the widespread adoption of these technologies for the creation of clinically viable scaffolds in real-world applications, particularly for bioprinting, relies on solving practical issues. These include balancing the universality and specificity of the currently limited selection of materials/bioinks for various sites and degrees of injury, achieving high-resolution printing with both speed and accuracy, enabling easy sterilization and off-the-shelf storage of scaffolds, and maintaining cell viability or bioactivity of incorporated substances [110]. To avoid slow degradation that inhibits tissue regeneration, materials need to be selected to match the rate of tissue regeneration. However, the degradation needs of materials vary for different sites and degrees of injury. Therefore, a wider range of material selection is needed to meet the complexity of native tissues and applicability to clinical injuries. Differences between acellular and cellular scaffolds are worth considering when designing gradient musculoskeletal scaffolds. While both need to fulfill biocompatibility requirements, there are significant differences in functionality, design considerations and application environments. Acellular scaffolds are engineered to possess specific mechanical strength and rigidity that closely mimic the native tissue they are intended to replace, ensuring proper load-bearing capabilities. In contrast, cellular scaffolds prioritize flexibility and a supportive microenvironment that accommodates cell growth and movement in addition to the above requirements. Acellular scaffolds must exhibit long-term biocompatibility by ensuring that they do not release toxic substances as they degrade, thus maintaining a safe environment for surrounding tissues. Cellular scaffolds additionally need to enhance cellular interactions, requiring surface modifications to promote effective cell adhesion, proliferation, and overall functionality. Cellular scaffolds typically demonstrate higher regeneration efficiency because they introduce exogenous cells to the damaged area and actively support cell behavior, such as migration and differentiation, which are crucial for tissue repair. Acellular scaffolds mainly provide structural support and may require additional factors or endogenous cells to stimulate regeneration. The degradation rate of both acelluar and cellular scaffolds should be carefully calibrated to match the pace of tissue regeneration, allowing for a seamless transition as new tissue forms. Cellular scaffolds should also be able to modulate the release of cells at the target site in a spatially and temporally controlled manner. Acellular scaffolds aim to provide stable biocompatible matrices that are endowed with specific functionality through structural design and loading of drugs and factors. Cellular scaffolds also involve the exogenous cells and their secretory factors to create an optimal regenerative environment and directly participate in tissue repair.

### Gradient Scaffolds Made by Sequential Layering of Hydrogels

Hydrogel materials used in tissue regeneration exhibit tissue-like properties mimicking the native ECM, including softness, inherent elasticity, and high water storage capacity. They can be made to exhibit minimal immunogenicity, controllable degradation rate, and excellent permeability, providing a conducive growth environment that promotes cell adhesion, migration, and repair function [111, 112]. As shown in Table 4, hydrogel materials used to construct gradient scaffolds are often manipulated by optimizing their composition in different layers, including through the incorporation of gradients of cells and/or bioactive substances during hydrogel formation, which is a unique capability compared to pre-fabricated scaffolds. The mechanical properties of different layers can also be modulated by controlling the strength of cross-linking during hydrogel formation [113], enabling the creation of anisotropic structures beneficial for the regeneration of mechanically graded tissues such as bone and interface tissues.

A direct method for fabricating the gradient scaffold using hydrogels is to layer sequential hydrogel formulations followed by cross-linking the hydrogels in different layers. In a recent study, a bilayered hydrogel scaffold was developed for osteochondral tissue regeneration based on sequential formulation and cross-linking of cartilage and bone layers [114]. The two layers presented a compositional gradient of methacrylated sodium alginate (SAMA), GelMA, and β-TCP of different proportions corresponding to cartilage and bone regeneration. The hydrogel layers were formed by photopolymerization of the C = C double bond in SAMA and GelMA, which could be triggered by blue light to form interpenetrated covalent hydrogel networks to avoid delamination of different layers of hydrogel. A biochemical gradient of KGN release within the chondral and osseous layers was also formed. A high concentration of KGN in chondral layer induced chondrogenesis of the embedded BMSCs, and a low concentration of KGN combined with β-TCP in the osseous layer promoted better osteogenesis compared to β-TCP only without KGN. The chondral and osseous layers also showed a gradient of pore sizes, transitioning from 150–200 μm in the top layer to 200–300 μm in the bottom layer, respectively, matching the requirements for chondrogenic and osteogenic differentiation. This hydrogel scaffold was found to promote superior repair in a rat osteochondral defect model, with the regenerated tissue showing a transition from hyaline cartilage to hypertrophic cartilage and calcified bone.

Using a similar design strategy of layering gradient hydrogels and incorporating bioactive molecules for osteochondral tissue engineering, a bilayer hydrogel was fabricated using a one-pot method, with two seamlessly integrated but distinct layers [115]. The upper layer comprised a GelMA-PDA hydrogel for cartilage repair, while the lower layer of GelMA-PDA/HAp hydrogel containing HAp nanoparticles was formed through PDA-induced in situ mineralization of calcium and phosphate ions. The bilayer hydrogel was formed by simultaneously polymerizing the two hydrogel layers, casting the lower layer followed by the upper layer. The high viscosity of the pre-gel solutions prevented the layers from fusing during polymerization. Moreover, TGF-β3 and BMP-2 were immobilized, respectively, in the cartilage and bone layer to help induce tissue-specific differentiation. Compared with pure GelMA hydrogel, the bilayer hydrogel induced better osteochondral tissue repair after implantation for 12 weeks in a rabbit full-thickness osteochondral defect model, suggesting that the bilayer design was more effective at promoting the regeneration of interfacial tissues.

Bilayer scaffolds with sequential layering of hydrogels aim to separately replicate the characteristics of the cartilage and subchondral bone layers, but may be limited by potential delamination between layers and lack of transitional area between tissue regions. A continuous gradient hydrogel was designed to mimic the anatomical, biological, and physicochemical transition between cartilage and bone in osteochondral tissue [116]. The hydrogel scaffold, composed of a continuous collagenous matrix presented a gradient distribution of HAp particles, resulting in a physical gradient of stiffness from the softer cartilage-like region to the stiffer bone-like region. The pores were open and interconnected within and between layers, contributing to the overall structure and mechanical properties of the scaffold. Biological evaluation using human BMSCs showed that the scaffold supported cell proliferation under both osteogenic and chondrogenic conditions, while its gradient of composition and stiffness preferentially directed cell growth in the cartilage and bone sub-regions.

**Fig. 4:**
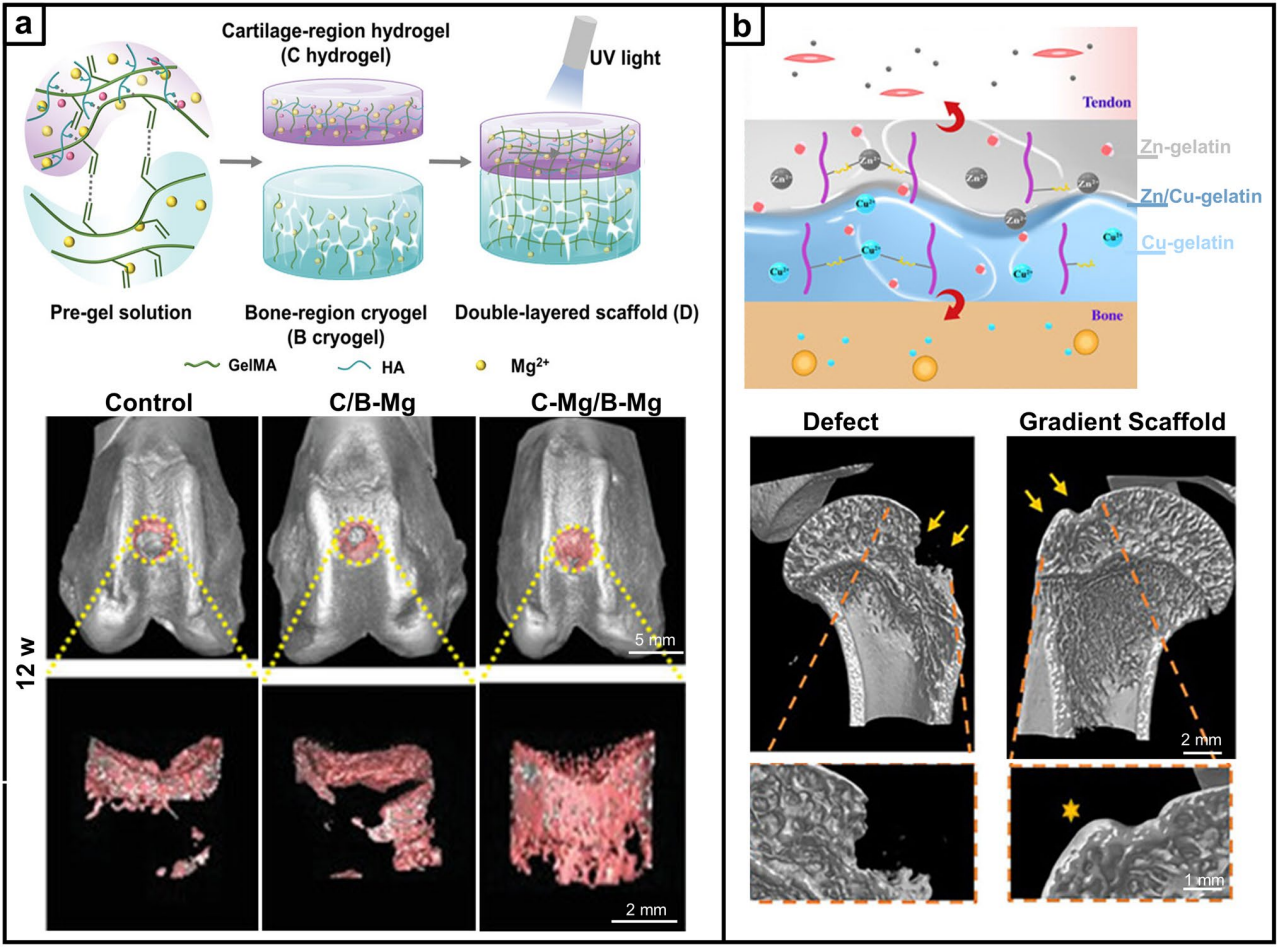
Gradient scaffolds prepared using hydrogel. **a** Preparation method of a bilayer gradient scaffold for osteochondral repair and reconstructed 3D micro-CT images at 12 weeks post-implantation (yellow circles denote the borders of original defects), C: GelMA and HA hydrogel, B: GelMA cryogels [119]. Copyright 2023, Wiley. **b** Mechanism of gradient bimetallic ion hydrogels in rotator cuff injury repair and micro-CT images of the defect area after eight weeks of implantation [120]. Copyright 2022, Royal Society of Chemistry

Other types of gradient hydrogel design approaches have made use of bioactive metal ions, including trace elements found in natural bone that have functions in promoting the regeneration of musculoskeletal tissues. For example, magnesium ions (Mg^2+^), zinc ions (Zn^2+^), and calcium ions can promote bone growth while copper ions (Cu^2+^) and cobalt ions can promote blood vessel growth [117]. For bone defects with osteoporosis, strontium ions can inhibit osteoclasts and promote osteogenesis. Additionally, some metal ions with antibacterial properties, such as silver and copper ions, can be used to treat bone defects with infection [118]. In one study, a bilayer hydrogel scaffold containing metal ions was designed to mimic the natural osteochondral structure (Fig. 4a) [119]. The upper layer consisted of GelMA and hyaluronic acid (HA), with small pores and a minor amount of magnesium carbonate hydroxide loaded in the hydrogel. The lower layer was formed by a GelMA solution loaded with a substantial amount of magnesium carbonate hydroxide and subjected to freeze-drying, resulting in a scaffold with larger pores. The release of small amounts of Mg^2+^ from the upper hydrogel layer promoted cartilage repair, while the long-term release of large amounts of Mg^2+^ from the lower freeze-dried gel enhanced mineralization and bone regeneration. The scaffold suffered 70% weight loss after 21 days in collagenase II solution (1 U mL^−1^). The upper layer of the scaffold showed a cumulative release of 200 ppm Mg^2+^ over 21 days, while 100 ppm Mg^2+^ was released from the lower layer of the scaffold. The lower layer showed very limited release of Mg^2+^ after day 7, while the upper layer maintained its initial release trend. In addition to its gradient composition, the gradient porosity along the scaffold resembled natural osteochondral tissue structure. Micro-CT imaging of different scaffold groups implanted in rabbit osteochondral defects for 12 weeks demonstrated that the bilayer hydrogel scaffold with magnesium ion gradients better facilitated simultaneous bone and cartilage regeneration. In another study, a layered hydrogel featuring a unique gradient distribution of copper and Zn^2+^ in a thiolate gelatin matrix was fabricated, mimicking the natural transition at the tendon-to-bone insertion site (Fig. 4b) [120]. As osteoblasts were more likely to be attracted to a copper-rich environment, while tenocytes were more likely attracted to a zinc-rich environment, the hydrogel had an increasing concentration of Zn^2+^ from the bone region up to the tendon region together with an opposite concentration gradient of Cu^2+^. The hydrogel precursors in the upper and lower regions were fused during fabrication through a one-step coordinative cross-linking process (the preparation of the scaffold through a coordinated cross-linking reaction completed in a single step), allowing the ions to form the two concentration gradients as well as an intermediate transition zone containing both Cu^2+^ and Zn^2+^. The different regions of the scaffold both degraded about 70% on day 21. Meanwhile, the cumulative release of Zn^2+^ was 75% and of Cu^2+^ was 72% on day 21. Since the same trend was observed in the ion release and degradation behavior, these two processes were likely to be occurring simultaneously. In vitro cultures indicated significantly elevated expression of COL III and scleraxis (SCX) by tenocytes in the Zn-rich tendon region, and of runt-related transcription factor 2 (RUNX2) and osteocalcin (OCN) by osteoblasts in the Cu-rich bone region at day 3 before the scaffold showed significant structural disruptions due to degradation. This simultaneous reparative effect for both tendon and bone was confirmed in vivo using a rat model of rotator cuff tear, where the gradient hydrogel scaffold showed better interface tissue regeneration compared to hydrogels with single metal ions after 8 weeks. The incorporation of Cu^2+^ and Zn^2+^ also conveyed an additional benefit of antibacterial properties, potentially providing a dual function of infection prevention and tissue regeneration in tendon-to-bone healing.

The gradient scaffolds prepared through hydrogels can be adjusted with different gel components, active drug types, and concentration gradients to form a multilayer or continuous composite gel structure, which fits the needs of complex bone interface repair. Also, buoyancy-driven gradients can be formed when two miscible and solidifiable liquid phases with a sufficient density difference are present. By introducing one liquid phase material into the other, the two phases establish a gradient over time, which can then be maintained by triggering a polymerization or gelation process. Molly et al. succeeded in achieving a concentration gradient for a variety of substances (GelMA, gellan gum, agarose, and acrylate polymers) by this method, whereby a gradient concentration of BMP-2 could be released over a 28-day period [121]. Glycosylated superparamagnetic iron oxide nanoparticles loaded with growth factors placed in agarose hydrogels were also able to form a concentration gradient of BMP-2 in the presence of magnetic field forces, which continued to release BMP-2 at 28 days [122]. It is important to note that the different layers of the hydrogel should ideally be able to react or have strong interactions to form physical or chemical cross-links or intermolecular forces such as hydrogen bonds to avoid delamination. Future studies can continue to explore the application of hydrogel scaffolds in other types of musculoskeletal and interface tissue regions, such as the intervertebral disk.

### Other Methods for Fabricating Gradient Scaffolds

The three main categories of electrospinning, additive manufacturing, and hydrogel layering described above represent the mainstream methods of preparing gradient scaffolds for regenerating musculoskeletal tissues. Other emerging approaches to scaffold preparation have attempted a combination of two or three methods to compensate for the shortcomings of each. For example, 3D rigid scaffolds with a gradient structure can be used to mimic cortical and cancellous bone tissues and injected internally with soft hydrogels to further impart ECM properties to the scaffolds [103]. Different fabrication methods can also be integrated to create innovative techniques that involve rotational gas foaming [123, 124], freeze-drying [125], and microfluidics [126]. For example, rotational gas foaming utilizes the gases produced by a reaction to expand the scaffold pores, thereby developing a 2D material into a 3D structure. In one study, a 3D nanofibrous scaffold with a structural gradient was fabricated by gradually reducing the amount of pluronic F-127 incorporated into the nanofibers in each successive layer [127]. The 2D nanofiber membranes were then converted into 3D assemblies exhibiting a gradient in pore sizes after the gas foaming expansion process, since each sequential layer expanded less than the previous layer. Another approach combined electrospinning with rotational gas foaming to fabricate scaffolds possessing diverse pore sizes or radial gradient structures, specifically designed for cranial bone regeneration [123]. Meanwhile, scaffolds with gradient structures can also be fabricated by freeze-drying and microfluidics through manipulating the growth of ice crystal and flow rate, respectively. For example, a scaffold with reverse opal structure was created using PLGA microspheres and HAp suspension [128]. The HAp was applied layer-by-layer with decreasing concentration from bottom to top. Subsequently, laser processing was used to generate parallel channels that mimicked the parallel arrangement of collagen fibers in natural tendons, resulting in a scaffold with gradient changes in both composition and structure. Another technique utilized layer-by-layer tape casting to create a composite film for creating a tendon-to-bone transition [129]. Four consecutive layers were constructed with varying ratios of PCL and calcium phosphate silicate, with increasing mineral content from the tendon region to the bone region, mimicking the natural tissue gradient. This composite film was found to improve tendon-to-bone integration in a rabbit model of supraspinatus tendon repair.

## Preclinical Performance of Gradient Scaffolds in Musculoskeletal Repair

Preclinical testing in animal models of musculoskeletal repair is a necessary step in the translation of innovative gradient scaffold designs into clinical application. The majority of scaffolds discussed in this review can be tailored to suit a wide range of animal species or defect sizes, such as rats, rabbits, goats, and horses. As seen in Table 1, nearly all studies that conducted in vivo testing of gradient scaffold designs noted significant reparative effects in bone, osteochondral, or tendon-to-bone injuries. While these findings suggest promise in future clinical use, it is imperative to recognize and record the constraints of testing in animals whose anatomy and physiology have distinct differences compared to humans. Certain limitations in the selection of animal models cannot be avoided. Most importantly, constrained by accessibility, study timeframe and cost, the vast majority of preclinical animal studies testing gradient scaffolds are conducted using small animals such as rats and rabbits that have a very short lifespan and different tissue healing capacities compared to humans, as well as young animals with skeletal structure and physiology that do not closely resemble elderly humans in whom musculoskeletal conditions are usually found. Larger-sized animals such as pigs and sheep, as well as aged animals are more anatomically and physiologically similar to humans with musculoskeletal conditions, but their use is greatly limited by cost, availability, and ethical concerns.

### Animal Model Sizes and Ages

All of the research discussed in this review that conducted in vivo testing of gradient scaffolds for musculoskeletal repair used small animals such as rats and rabbits. The studies that used rats [103, 105, 106, 114, 116, 130] included different strains of laboratory rats, such as female Lewis rats (16 weeks old, average weight 454 g) [106], female Fischer rats (12 weeks old, average weight 245 g) [116], and male Sprague–Dawley rats (300 g) [103]. These ages correspond to adolescence or early adulthood in human years, when these young animals exhibit superb self-healing capacity in addition to the naturally superior healing ability of prey species. Similarly, studies that used New Zealand white rabbits [70, 89, 92, 94, 115, 131–135] to test gradient scaffolds involved young animals typically prior to or at sexual maturity (5–7 months). For instance, studies on bone regeneration have used rabbits that were aged 3 months [131] and 6 months [133] while studies on osteochondral and tendon-to-bone regeneration have used rabbits aged 3 months [135] and 5 months [129], respectively. However, in humans, the incidence of fractures [136], osteoarthritis [137], and rotator cuff tears [138] increases dramatically above 65 years of age. It is also known that as the human body ages, progenitor cells normally responsible for musculoskeletal tissue repair exhibit reduced numbers and regenerative capacity, frequently contributing to the impaired healing outcomes seen in elderly individuals [139]. Therefore, using young laboratory animals resembling human adolescence and early adulthood is an inherent limitation, as they model a period of time characterized by excellent self-repairing capacity and a relatively low prevalence of musculoskeletal diseases, with likely different cellular and molecular mechanisms governing the repair of bone and related tissues compared to aged animals with diminished regenerative potential. Moreover, animal models of musculoskeletal injury are often treated at the time of surgical defect creation, which does not accurately resemble clinical scenarios whereby injuries have often progressed for some time, frequently into chronic injuries before treatments are applied. These factors should be considered when interpreting the positive outcomes of regeneration obtained using gradient scaffolds in animal models of musculoskeletal injuries, which also call for future investigations using more physiologically relevant models such as in aged animals and chronic defects.

### Comparison with Other Scaffolds Tested in Large Animal Models

**Fig. 5:**
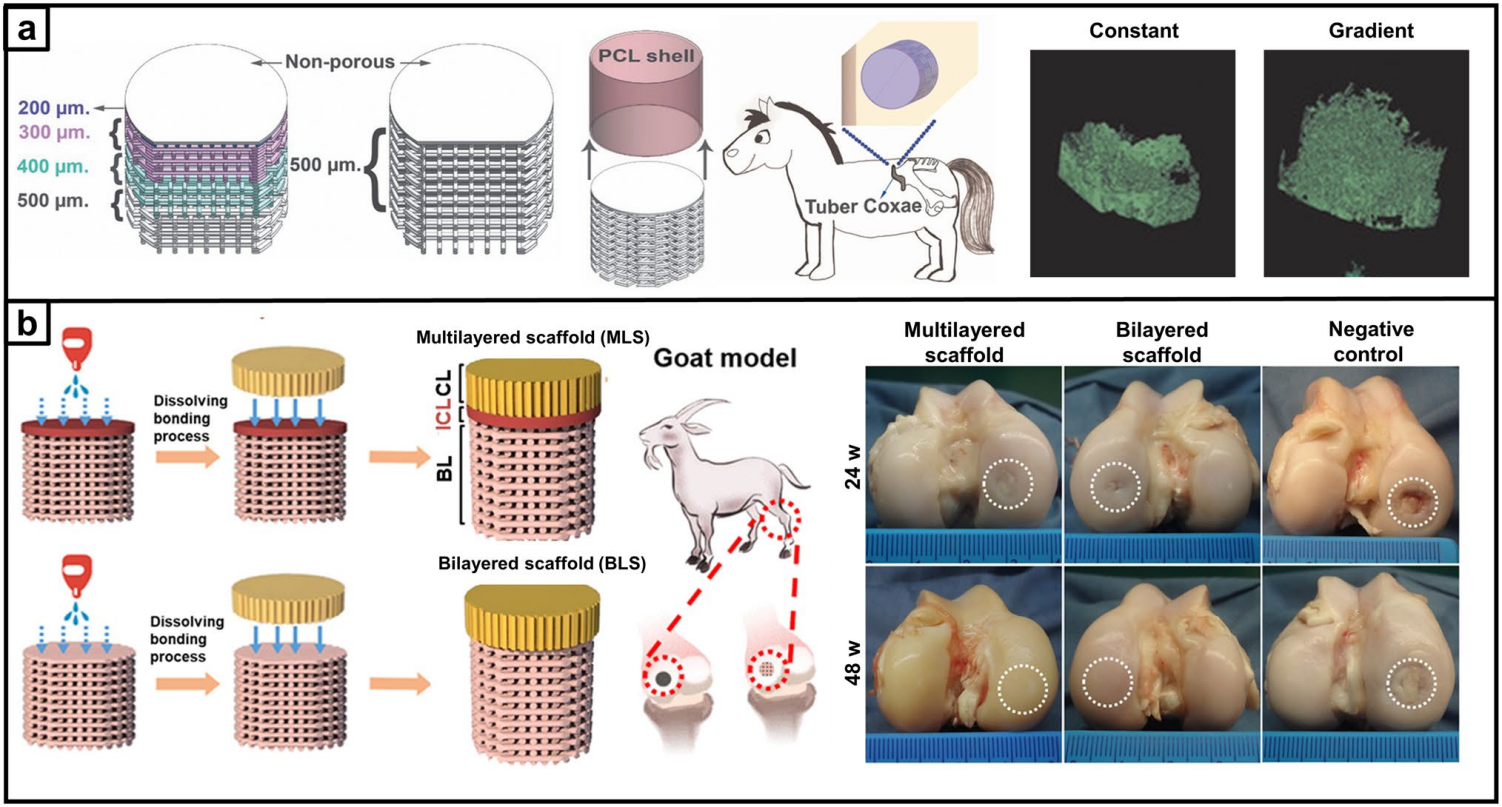
Application of scaffolds design in large animal models. **a** 3D pore size gradient scaffold applied to bone defects in horses and 3D reconstructed µ-CT images after 7 months of implantation of scaffolds with constant and gradient porosity [141]. Copyright 2019, Wiley. **b** Multilayered scaffold made by 3D printing for osteochondral of goats and gross morphology of osteochondral defect repair at 24 and 48 weeks (BL: bone layer; ICL: intermediate compact layer; CL: cartilage layer) [143]. Copyright 2018, American Chemical Society

Although the studies discussed in this review have not tested gradient scaffold designs using large animal models of musculoskeletal repair, other types of scaffold implants have been examined in sheep [140], horses [141] and goats [142, 143]. For example, a porous calcium phosphate bioceramic scaffold with 3D printed layers that had varied pore size between layers (500, 400, 300, and 200 µm) was compared to a scaffold with constant 500 µm pore size to repair critical-sized bone defects in horses (Fig. 5a) [141]. The scaffolds were implanted into the ilium of horses (aged 5–9 years, weight 275–375 kg) for 7 months. The scaffolds with constant porosity showed significantly lower total new bone formation and scaffold degradation compared to gradient porosity scaffolds, which achieved an enhanced degree of bone regeneration and remodeling. In another study, experiments were conducted using a porous multilayered titanium alloy implant with pore sizes ranging from 300 to 400 μm in osteochondral defects of mature goats with an average weight of 45 ± 5 kg (Fig. 5b) [143]. Experimental results at 24 and 48 weeks post-implantation indicated that the multilayered scaffold was more effective at promoting defect repair compared to bilayered scaffold and blank groups. The findings suggested that under simulated physiological loads, multilayered implants could provide early load-bearing capacity and effectively enhance bone integration. Given that this animal model closely approximated human adult bone structure and weight, the study results were valuable in guiding the treatment of clinically relevant large bone defects. There is a lack of evidence for the outcomes of scaffold-based repair of the tendon–bone interface in large animals.

These studies illustrate that musculoskeletal repair in large animals, which more closely represent human skeletal structure and physiology, may proceed differently compared to the results seen in smaller animals. Again, this stresses the importance of verifying new biomimetic scaffold designs in physiologically relevant large animal models prior to considering clinical applications. For modeling musculoskeletal injuries, large animals such as sheep, goats, horses, pigs, and dogs have greater anatomical and physiological similarities to humans. This is reflected both in the size scales of tissues, allowing sufficient space for defect creation that mimics the defect and scaffold sizes expected in humans, and also in the rate of tissue metabolism and progression of tissue repair in response to injury. The longer lifespan of large animals makes them suitable for conducting longitudinal studies and observing the long-term effects of treatments. In summary, it is essential to consider both the benefits and drawbacks when selecting specific animal models to test gradient scaffolds for applications in musculoskeletal repair. Despite the advantages of larger animals, these need to be balanced with their limitations in accessibility, cost, space requirements, and ethical concerns. Non-human primate models may be considered in late-stage preclinical investigations to more accurately mimic the clinical setting [144].

## Conclusions and Future Perspectives

Considering the existence of multiple gradients in musculoskeletal tissues including variations in structure, biochemical composition, mechanical properties, and cellular phenotype, gradient scaffolds have significant potential in achieving faithful regeneration of bone and interfacial skeletal tissues. In this review, we have summarized and critically analyzed gradient scaffolds that have been developed to regenerate three main types of musculoskeletal tissues: bone, osteochondral tissue, and tendon-to-bone interface. We highlighted the interesting design features seen in recent studies and reported the advanced manufacturing strategies used to create these gradient scaffold designs, including electrospinning, additive manufacturing, and hydrogel fabrication techniques. Our review points to the advantages of using synergistic techniques and integrated approaches for producing gradient scaffolds, to more effectively replicate native hierarchical tissue structure and musculoskeletal tissue repair outcomes. Current preclinical investigations in small animal models indicate promise in the ability of gradient scaffold designs to improve the future treatment of musculoskeletal injuries.

Despite rapid developments in gradient scaffold designs for musculoskeletal repair in recent years, a number of challenges remain to be addressed before they may attain wider clinical applicability. Firstly, possibly limited by the available choices for fabrication techniques, current gradient scaffolds do not faithfully replicate all of the features of native bone and interface tissues. Most scaffold designs focus on biomimicry at the macroscopic tissue level, but fail to provide mimicry for sub-structural tissue units. In addition, current gradient scaffolds could benefit from a better match with the gradients of ECM and cell distributions found in native tissues, as many designs still exhibit sharp borders between scaffold layers rather than a smooth transition. Even for scaffolds with smooth gradient transition of properties, the majority show gradient lengths that span hundreds of micrometers to even millimeters, which is wildly out of proportion compared to the size of native tissue gradients. In the future, gradient scaffolds with biomimetic design and length scales may be produced using techniques that provide increased control and precision, such as near-field electrospinning [145].

Secondly, enhancing the ability of gradient scaffolds in the modulation of cell behavior toward musculoskeletal repair is an important development direction. Many studies have introduced various types of bioactive substances, such as growth factors, bioactive peptides, or immunomodulatory molecules into gradient scaffolds to help regulate cell behavior, but the establishment of functionally useful biochemical gradients and the dosage of biomolecules require further exploration. In this regard, it is important to understand how degradation affects the gradient structure or composition of a scaffold, and consider this factor in scaffold design such that the rate of degradation matches the progress of tissue regeneration. During the degradation of a gradient scaffold, the gradient structure begins to disappear and the scaffold components are not permanently retained in the body. The retention time (or degradation time) of a gradient varies depending on the chemical composition, structure, and even the preparation method of the scaffold. For example, an electrospun SF scaffold underwent degradation in 1 U mL^−1^ protease XIV solution with a mass loss of 65% after 24 days [146]. In another study, SF scaffolds prepared by electrospinning and freeze-drying also degraded gradually over time when immersed in 5 mL of protease XIV in PBS (2 mg L^−1^) solution, reaching a plateau at 11.86% degradation after 20 days [46]. Scaffolds made of synthetic polymers tend to exhibit slower degradation than those comprising natural polymers, and their degradation rate may also be modulated by composition. For example, poly[(rac-lactide)-co-glycolide] (85:15) scaffolds have a degradation time of 5–6 months, while poly[(rac-lactide)-co-glycolide] (50:50) scaffolds degrade in 1–2 months [147]. Specific surface area, which is associated with scaffold porosity, has a significant effect on degradation rate, where higher ratios lead to faster dissolution. For example, PCL scaffolds with 90% porosity degraded by 50% at 72 weeks in vitro, while PCL with 80% porosity degraded by only 10% [148]. However, it is interesting to note that the time taken for scaffolds to start regulating cell behavior may be much shorter than the time required for significant degradation. In one study, a hierarchically degradable bioactive bone scaffold was constructed by adjusting the ratio of hydrogel material (polyethylene glycol/GelMA) added with decellularized bone matrix (DBM) particles and BMP-2 [149]. First, the degradation of DBM left inward growth channels for new tissues and capillaries. The inward growth of new tissues and capillaries then propelled secondary degradation of the scaffold. The scaffold had degraded about 40%–60% at day 28, matched by a similar trend of BMP-2 release from its different layers. Remarkably, the scaffold showed very early stage effects on cellular osteogenic differentiation. Alkaline phosphatase (ALP) staining in the scaffold at day 3 was significantly higher than that of the negative control. Gene markers of osteogenesis such as RUNX2 and OCN also showed significant and early upregulation in scaffolds over the period of differentiation. Unfortunately, current studies usually only explore the overall degradation rate of scaffolds in vitro and rarely report the changes in gradient properties during scaffold degradation. This is an issue that warrants investigation in future studies. In addition, current evidence suggests that the micro- and nano-morphology of scaffolds can reciprocally interact with mechanical conditioning, protein adsorption, and immunomodulation among other pathways to regulate cell behavior [150, 151]. Future gradient scaffold design strategies may benefit from integrating the control of micro- and nanoscale topography with mechanical stimulation to enhance musculoskeletal tissue regeneration, particularly considering that the functional role of these tissues is closely regulated by movement and force.

Thirdly, considering that the natural progression of in vivo tissue healing and regeneration involves a complex and long-term process, new gradient scaffold design need to meet the demands of different phases during musculoskeletal tissue healing. The development of stimulus-responsive materials and their incorporation into “smart” scaffolds [152, 153] coupled with the construction of biochemical, mechanical, or other gradients may help to achieve more precise regulation of cellular behavior and consequently the biological cascades essential for long-term healing. The microenvironment in which tissue healing occurs can also vary considerably among patients depending on the physical condition of individual, area of damage/disease, and intrinsic repair capacity, among other factors. The development of personalized scaffolds constitutes an important step in satisfying the clinical need to cater for variations in patient characteristics, which may benefit from rapid developments in artificial intelligence and medical imaging to assist scaffold design. For instance, high-resolution imaging can be used to capture precise and individualized information on musculoskeletal tissue defects to enable the production of customized scaffolds, while artificial intelligence may be employed to efficiently compute optimal scaffold design parameters to simultaneously satisfy multiple design requirements [154–157].

Last but not least, there are significant problems to consider in the large-scale manufacturing and scale-up of current gradient scaffold design strategies. Highly biomimetic scaffold designs typically involve a series of complicated fabrication steps that create barriers to efficient and replicable production on a commercializable level. There are also technical barriers to creating gradient scaffold structures that are highly precise, nonlinear, or contain a mixture of composite materials. The most popular methods for fabricating gradient scaffolds currently involve hydrogel materials, drawing from a limited selection of materials that may also pose the issue of mismatch between scaffold degradation rate and tissue regeneration rate, as well as the accumulation of degradation products with adverse effects [51]. For musculoskeletal tissue regeneration, hydrogel materials typically also exhibit weak mechanical properties that are not suitable for load-bearing applications. It is important to note here that although a range of animal models have been used in the literature to probe the in vivo outcomes of musculoskeletal repair using gradient scaffolds, there are fundamental differences between the anatomical and biomechanical characteristics of the skeletal system in animals compared to humans, and these differences need to be carefully considered when interpreting the findings before potentially translating the scaffolds to clinical application [12, 158]. The field of musculoskeletal regenerative medicine and development of gradient implants would benefit from improved standardization of the industry framework for evaluation and translation. For instance, the development of standardized preclinical evaluation methods and uniform evaluation criteria will improve the consistency of research findings and accelerate the commercialization of new discoveries [159]. With the integration of sophisticated and biomimetic scaffold designs, advanced manufacturing strategies, and standardization of steps to clinical translation, we can anticipate gradient scaffolds to have a significant contribution toward clinical applications in the treatment of challenging musculoskeletal injuries and diseases.
